# Differential condensation of FLO6 influences grain amylose synthesis in rice

**DOI:** 10.1126/sciadv.aec8768

**Published:** 2026-08-14

**Authors:** Fei Liu, Minghao Zhang, Zhipeng Yan, Yifei Huang, Hui Zhang, Jing Zhang, Kabin Xie, Yibo Li, Yuqing He, Deqiang Duanmu, Qiang Wang, Jean-David Rochaix, Min Ouyang

**Affiliations:** ^1^ College of Life Science and Technology, Huazhong Agricultural University, Wuhan, Hubei 430070, China.; ^2^ School of Life Sciences, Hubei University, Wuhan 430062, China.; ^3^ Shanxi Hou Ji Laboratory, College of Life Sciences, Shanxi Agricultural University, Jinzhong, Shanxi 030801, China.; ^4^ National Key Laboratory of Crop Genetic Improvement, Huazhong Agricultural University, Wuhan, Hubei 430070, China.; ^5^ State Key Laboratory of Agricultural Microbiology, College of Life Science and Technology, Huazhong Agricultural University, Wuhan 430070, China.; ^6^ State Key Laboratory of Crop Stress Adaptation and Improvement, School of Life Sciences, Henan University, Kaifeng 475004, China.; ^7^ Department of Molecular Biology, Department of Plant Biology, University of Geneva, Geneva, Switzerland.

## Abstract

Amylose content is widely regarded as one of the decisive parameters determining rice grain quality. However, the regulatory mechanisms underlying amylose synthesis across different rice subspecies remain poorly understood. Here, we report that FLO6 (Floury Endosperm 6) undergoes phase separation to form condensates that adhere to the surface of starch granules, and these condensates are capable of recruiting GBSS1 (Granule-Bound Starch Synthase 1). A single-nucleotide polymorphism (SNP^3194^) within the *FLO6* gene endows the *FLO6* alleles from *japonica* and *indica* subspecies with distinct condensate properties. This divergence results in varying GBSS1 activities within the FLO6 condensates, ultimately contributing to differences in grain amylose content. While the well-known allelic variation of *GBSS1* establishes the baseline enzyme abundance, the evolutionary adaptation of the FLO6-GBSS1 module serves as an essential superimposed regulatory layer that fine-tunes amylose synthesis across different rice subspecies. Our findings offer potential biotechnological avenues for directionally altering grain amylose content.

## INTRODUCTION

Through photosynthesis, plants synthesize starch, which is an efficient energy storage form composed of α-glucose, typically accumulated in various plant organs. For instance, the cereal endosperm serves as a major source of storage starch (*1*). This carbohydrate not only fuels the plants’ own growth and ensures their survival but also serves as a fundamental energy source that underpins the existence and development of human society. Compared to advancements in rice grain yield, improvements in grain quality have lagged behind, primarily due to the complexity of starch synthesis processes.

Amylose content plays a pivotal role in determining rice grain quality (*2*, *3*). Rice generally contains two types of Granule-Bound Starch Synthase, GBSS1 and GBSS2, with GBSS1 specifically functioning in grain amylose synthesis (*4*). Encoded by the *Wx* gene, GBSS1 was first isolated in 1990 (*5*). In recent years, numerous allelic variants of *Wx* have been identified, such as *Wx^a^*, *Wx^b^*, *Wx^in^*, and *Wx^op^*, and these four alleles can be distinguished by six functional polymorphic sites (*6*). *Wx^a^* mainly exists in the *indica* rice cultivars and is related to high amylose content of rice grain. In contrast, *Wx^b^* is mainly distributed in the *japonica* rice cultivars and is responsible for low to intermediate amylose content. This difference is primarily due to a guanine-to-thymine mutation at the 5′ splicing site of intron 1 in the *Wx^b^* allele, which impairs mRNA splicing and leads to much lower GBSS1 protein levels in *japonica* rice (*6*, *7*). Notably, at the protein level, the mature GBSS1^a^ and GBSS1^b^ differ by only a single amino acid (*6*, *7*). Besides grain amylose content, these variations further influence eating and cooking quality, gel consistency, gelatinization temperature, and grain viscosity (*8*). Genomic modifications of *Wx* using editing techniques have proven effective in fine-tuning amylose content to enhance rice grain quality (*9*–*13*). While such modifications artificially alter GBSS1 activity, the intrinsic regulatory mechanisms governing GBSS1 activity during starch synthesis in rice grains remain poorly understood.

FLO6 (Floury Endosperm 6) was first reported in 2014 as a non-enzymatic factor regulating starch synthesis in rice grains (*14*). Loss of FLO6 function results in a floury endosperm phenotype, characterized by reduced starch and amylose content, along with altered physicochemical properties of starch granules (*14*). FLO6 and its orthologs, such as *Arabidopsis* PROTEIN TARGETING TO STARCH 2 and wheat B-GRANULE CONTENT 1, have all been implicated in starch granule initiation in either leaf or endosperm tissues (*14*–*16*). FLO6 has been shown to interact with isoamylase 1, starch synthase IVb, and GBSS1 in rice grains (*14*, *17*). Because GBSS1 lacks a carbohydrate-binding module (CBM), it relies on CBM-containing proteins, such as the previously characterized PROTEIN TARGETING TO STARCH 1 (PTST1) and FLO6, to associate with starch granules (*3*). Thus, FLO6 may act as a scaffolding protein, regulating the binding of its interacting partners to starch via the carbohydrate-binding module 48 (CBM48) located at its C terminus (*14*). However, the mechanism by which FLO6 controls the activity of these enzymes to regulate starch synthesis is still unclear and remains elusive.

Here, we report that FLO6 undergoes liquid-liquid phase separation (LLPS) in rice Nipponbare (NPB), relying on its second intrinsically disordered region (IDR). Disruption of FLO6 phase separation impairs amylose synthesis in NPB grains. A conserved single-nucleotide polymorphism (SNP^3194^) located within the second IDR affects the condensate properties of FLO6 between *japonica* and *indica* subspecies. This change in FLO6 condensate properties may affect amylose synthesis by differentially recruiting GBSS1 and regulating its activity in rice grains. Base editing of SNP^3194^ in *FLO6* effectively alters amylose synthesis in NPB grains. It is plausible that SNP^3194^ variations among *FLO6* alleles in wild and cultivated rice adjust the mobility of FLO6 condensates, facilitating local adaptation to environmental changes during rice evolution in coordination with the evolution of GBSS1. Our results suggest a regulatory mechanism for amylose synthesis mediated by FLO6 through differential phase separation, and point to a potential strategy to directionally alter grain amylose content by manipulating FLO6 condensate properties.

## RESULTS

### GBSS1^b^-interacting protein FLO6*^jap^* undergoes LLPS in rice

Although GBSS1 is considered as the vital starch synthase functioning in amylose synthesis in rice grains, the regulation of its activity is still elusive. To elucidate the regulatory mechanism on GBSS1 activity, a yeast two-hybrid screen of a rice NPB (*Oryza sativa* L. ssp. *japonica*) seedling cDNA library (*18*) using GBSS1^b^ (encoded by *Wx^b^*, LOC_Os06g04200) as the bait was carried out to identify potential interacting partners and identified FLO6*^jap^* (LOC_Os03g48170) (Fig. 1A), which was first reported as a non-enzymatic protein participating in starch synthesis in the endosperm of NPB grains (*14*). The direct physical interaction between GBSS1^b^ and FLO6*^jap^* was also confirmed by the microscale thermophoresis (MST) assay (Fig. 1B and fig. S1A).

**Fig. 1.**
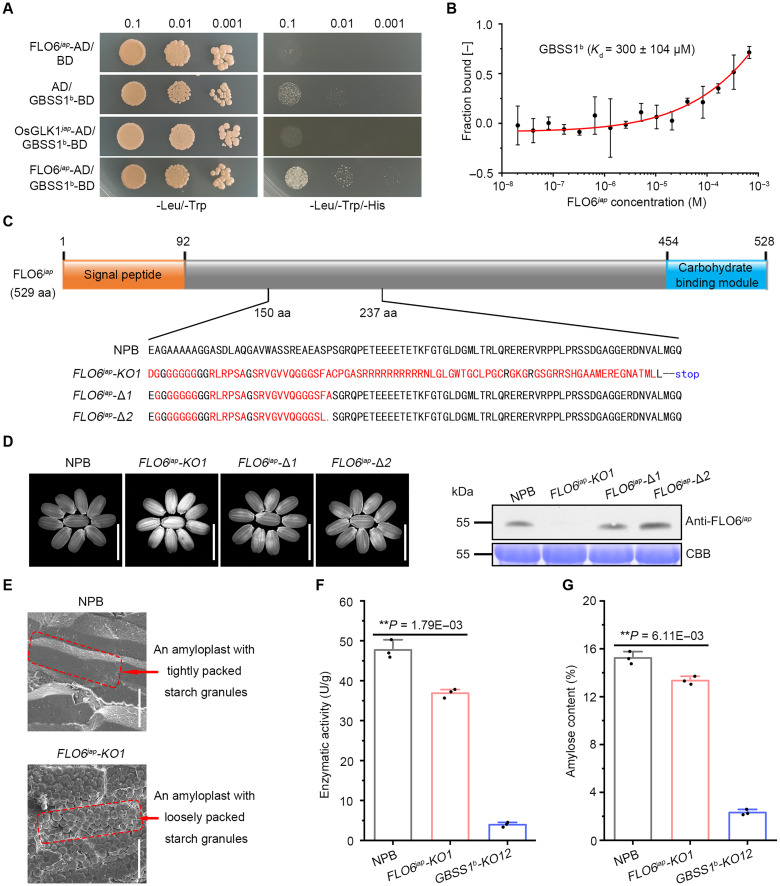
FLO6 *^jap^* interacts to GBSS1^b^ and regulates grain amylose synthesis in rice NPB. (**A** and **B**) Yeast two-hybrid and MST assays were used to assess the interaction between GBSS1^b^ and FLO6*^jap^*. Values are means ± SD (*n* = 3) in (B). AD, activation domain; BD, binding domain; CBB, Coomassie brilliant blue. (**C**) Schematic view indicating allelic information for FLO6*^jap^* in NPB and *FLO6* mutants. The “.” symbol indicates an amino acid position that is deleted relative to wild-type and the other lines. (**D**) Representative grains from NPB and *FLO6^jap^* mutants. Scale bars, 1 cm. The protein levels of FLO6*^jap^* determined by immunoblotting are listed on the right. Coomassie blue staining was used to check for equal loading of samples. (**E**) Scanning electron microscopy (SEM) analyses of the endosperm of NPB and *FLO6^jap^-KO1* grains. Scale bars, 50 μm. (**F** and **G**) Activity of GBSS1^b^ in the 15-DAP endosperm, and amylose content from NPB and *FLO6^jap^-KO1* and *GBSS1^b^-KO12* grains. Values are means ± SD (*n* = 3). The asterisks indicate statistical significance determined by a Student’s *t* test (**P < 0.01). Values of *GBSS1^b^-KO12* grains (fig. S1, C and D) are used as internal control to evaluate the accuracy of methods. aa, amino acid.

To further evaluate the influence of FLO6*^jap^* on GBSS1^b^ activity in rice grains, the CRISPR-Cas9 genome editing method was used to create *FLO6* knockout (KO) mutants in NPB. Through nonhomologous end joining repair, three independent homozygous mutants with distinct exonic indels were generated. *FLO6^jap^*-*KO1* contains two separate 1–base pair (bp) insertions that cause a frameshift and introduce a premature stop codon (Fig. 1C and fig. S1B). In contrast, *FLO6^jap^*-Δ*1* and -Δ*2* carry specific combinations of short nucleotide insertions and deletions (+1/−1 bp and +1/−4 bp, respectively). These paired indels induce localized frameshifts that alter stretches of 25 and 24 amino acids (from residues 151 to 180), respectively, before the original reading frame is restored (Fig. 1C and fig. S1B). Appearance of grains from *FLO6^jap^*-Δ*1* and -Δ*2* were similar to NPB grain, except for small areas of chalkiness (Fig. 1D), while *FLO6^jap^-KO1* grains had an obvious floury endosperm, round and loosely packed starch granules compared with NPB (Fig. 1, D and E), which is in line with the reported phenotype of *flo6* grains (*14*). Next, we used *FLO6^jap^-KO1* for further research because of its marked changes in grain phenotype. Notably, GBSS1^b^ activity in developing endosperm, and amylose content of rice grains were substantially decreased in *FLO6^jap^-KO1* compared to those of NPB, whereas *GBSS1^b^* KO mutant in the NPB background (*GBSS1^b^-KO12*), serving as a control, exhibited no measurable amylose (Fig. 1, F and G, and fig. S1, C and D). These findings suggest that FLO6*^jap^* might regulate GBSS1^b^ activity and amylose synthesis in rice grains.

To further dissect the function of FLO6*^jap^*, we first analyzed the subcellular localization of FLO6*^jap^*. Numerous punctate green fluorescence signals of FLO6*^jap^*–green fluorescent protein (GFP) emerged in chloroplasts under continuous dark conditions (Fig. 2A), indicating that FLO6*^jap^*-GFP might undergo phase separation in planta. To test this possibility, FLO6*^jap^* and FLO6*^jap^*-GFP were expressed and purified in vitro (fig. S2A). Considering that the physiological pH of amyloplasts is ∼7.0 to 8.0, all in vitro phase behavior assays in this study were conducted at both pH 6.5 and 7.5. Because FLO6*^jap^* and all other variants evaluated in this study exhibited highly consistent condensation behaviors and fluorescence recovery after photobleaching (FRAP) dynamics across both pH conditions (figs. S2 and S3), their in vitro properties are described collectively below.

**Fig. 2.**
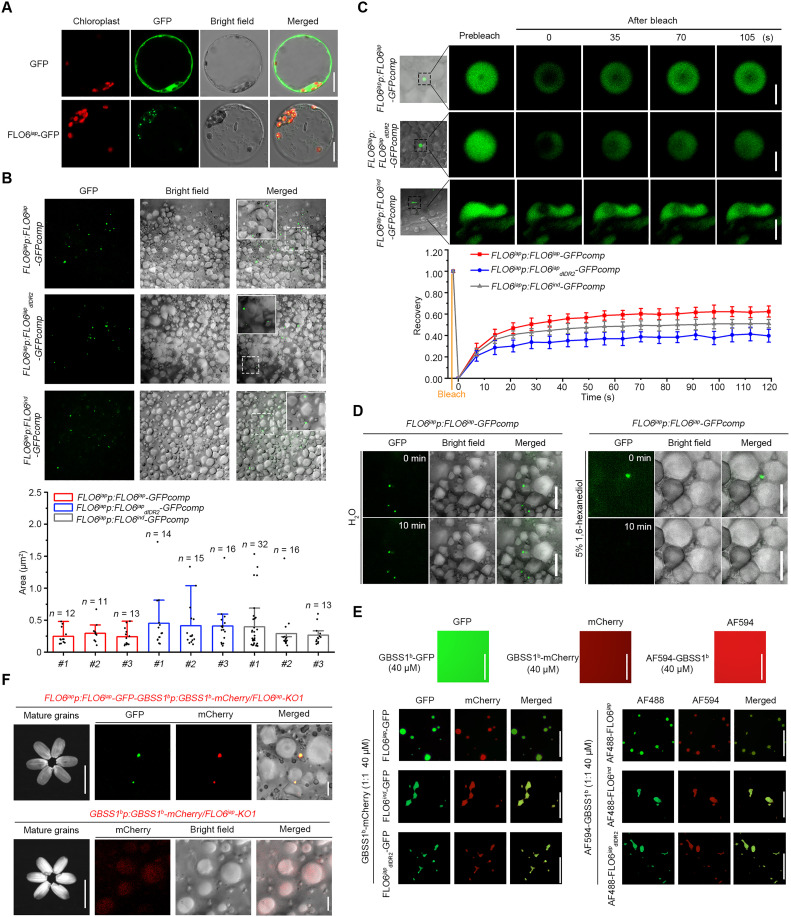
FLO6 alleles show different phase separation properties in vivo. (**A**) Fluorescence microscopy of NPB mesophyll protoplasts expressing FLO6*^jap^*-GFP under continuous dark conditions. Scale bars, 10 μm. (**B**) Quantification analysis of FLO6*^jap^*-GFP, FLO6*^ind^*-GFP, and FLO6*^jap^*_dIDR2_-GFP condensate properties in 15-DAP endosperm from the indicated transgenic plants. Scale bars, 20 μm. Enlargements of the representative localization regions are displayed in the upper corner of the pictures. Three different seeds were used to calculate the number and area of condensates. *n* indicates the condensate number. (**C**) FRAP of FLO6*^jap^*-GFP, FLO6*^ind^*-GFP, and FLO6*^jap^*_dIDR2_-GFP condensates in the 15-DAP endosperm from the indicated transgenic plants. Picture of photobleached condensate in bright field is displayed. Scale bars, 2 μm. Plot of fluorescence intensity recovery is shown below. Values are means ± SD (*n* = 10). (**D**) 1,6-Hexanediol treatment of FLO6*^jap^*-GFP condensate of the 15-DAP endosperm from *FLO6^jap^p:FLO6^jap^-GFPcomp* grains. Scale bars, 10 μm. (**E**) Phase separation of GBSS1^b^ labeled with different fluorescent tags and dyes, and in vitro recruitment assays of purified FLO6 (wild-type and variants) with GBSS1^b^. For recruitment, GFP-tagged FLO6 variants (retaining the 6 × His tag) were incubated with GBSS1^b^-mCherry, or AF488-labeled FLO6 variants were incubated with AF594-GBSS1^b^. The 6 × His tags were cleaved from all GBSS1^b^ proteins and dye-labeled FLO6 proteins, while GFP-tagged FLO6 variants retained the 6 × His tag. All proteins were used at 40 μM. AF488/AF594, AbFluor 488/AbFluor 594. Scale bars, 50 μm. (**F**) FLO6*^jap^* governs the spatial organization of GBSS1^b^ into condensates in vivo. Subcellular localization of GBSS1^b^-mCherry in 15-DAP endosperms from *GBSS1^b^p:GBSS1^b^-mCherry/FLO6^jap^-KO1* (absence of FLO6*^jap^*) and *FLO6^jap^p:FLO6^jap^-GFP-GBSS1^b^p:GBSS1^b^-mCherry/FLO6^jap^-KO1* (presence of FLO6*^jap^*) transgenic plants. Scale bars, 1 cm (left); 5 μm (right).

Numerous liquid droplet condensates appeared when FLO6*^jap^* and FLO6*^jap^*-GFP were diluted with buffer to a final NaCl concentration of 100 mM under both pH conditions (fig. S2B). Phase separation of FLO6*^jap^*-GFP was lost when the protein concentration of purified FLO6*^jap^*-GFP was below 5 to 10 μM (fig. S2C), indicating that phase separation of FLO6*^jap^* is concentration dependent. FRAP showed that the fluorescence signal of FLO6*^jap^*-GFP and FLO6*^jap^* condensates was recovered similarly at both pH 6.5 and 7.5 to a plateau of about 60% of the original level within 1 min after bleaching (fig. S2, D and E). This ∼60% recovery plateau indicates a partially immobile fraction within the condensates. In addition, the FLO6*^jap^*-GFP condensates readily collapsed into bigger droplets when contacting each other (fig. S2F), indicating that the FLO6*^jap^*-GFP condensates exhibit behaviors of liquid droplets in vitro despite partial aggregation. Besides, phase separation of FLO6*^jap^*-GFP was sensitive to NaCl and 1,6-hexanediol treatment (fig. S2, G and H), which indicates that hydrophobic and multivalent electrostatic interactions drive phase separation of FLO6*^jap^*.

To examine whether FLO6*^jap^* phase separates in vivo, we generated transgenic plants expressing *FLO6^jap^-GFP* driven by the native *FLO6^jap^* promoter in the *FLO6^jap^-KO1* (designated as *FLO6^jap^p:FLO6^jap^-GFPcomp*). Microscopy results showed that punctate green fluorescence signals of FLO6*^jap^*-GFP adhered to starch granules in *FLO6^jap^p:FLO6^jap^-GFPcomp* grains (Fig. 2B). FRAP assays showed that FLO6*^jap^*-GFP condensates in the endosperm display fluorescence recovery similar to that of FLO6*^jap^*-GFP in vitro (including the incomplete recovery plateau) (Fig. 2C). Furthermore, the FLO6*^jap^*-GFP puncta were sensitive to 1,6-hexanediol treatment, which resulted in the rapid elimination of the fluorescence signals within 10 min compared to the water control (Fig. 2D). These results collectively indicate that FLO6*^jap^* forms liquid-like phase-separated condensates in rice grains.

Considering the interaction between FLO6*^jap^* and GBSS1^b^, we examined whether FLO6*^jap^* condensates can recruit GBSS1^b^. Results demonstrated that although GBSS1^b^ is incapable of phase separation on its own, it can be recruited into FLO6*^jap^* condensates in vitro (Fig. 2E and figs. S1A and S2A). To determine whether the recruitment governs the spatial organization of GBSS1^b^ in vivo, we compared the localization of GBSS1^b^ in the presence and absence of FLO6*^jap^*. We generated transgenic lines expressing GBSS1^b^-mCherry and/or FLO6*^jap^*-GFP driven by their own native promoter respectively in the *FLO6^jap^-KO1* mutant, resulting the *GBSS1^b^p:GBSS1^b^-mCherry/FLO6^jap^-KO1* and *FLO6^jap^p:FLO6^jap^-GFP-GBSS1^b^p:GBSS1^b^-mCherry/FLO6^jap^-KO1* transgenic plants. We found that in the absence of FLO6*^jap^*, GBSS1^b^-mCherry was diffusely distributed around the starch granules. In contrast, the presence of FLO6*^jap^*-GFP orchestrated the coalescence of GBSS1^b^-mCherry into distinct phase-separated condensates on the starch granule surface (Fig. 2F). These in vivo genetic results indicate that FLO6*^jap^* is crucial for the accurate localization of GBSS1^b^ during the development of starch granule. Together, our findings demonstrate that FLO6 condensates recruit GBSS1^b^ both in vitro and in vivo.

### IDR2 is crucial for FLO6*^jap^* phase separation and amylose synthesis in rice grains

FLO6*^jap^* harbors two IDRs, which are termed IDR1 (133 to 262 amino acids) and IDR2 (375 to 418 amino acids) (fig. S3, A and B). To determine the role of IDRs in FLO6*^jap^* phase separation, IDR deletion assays were performed (fig. S3, B and C). Deletion of IDR1 (termed FLO6*^jap^*_dIDR1_) did not affect the formation of droplets compared to FLO6*^jap^*, and FRAP assays showed that the recovery efficiency of FLO6*^jap^*_dIDR1_ was similar to that of FLO6*^jap^*-GFP (figs. S2, D and E, S3, C and D). However, deletion of IDR2 (termed FLO6*^jap^*_dIDR2_) led to the formation of irregular condensates (fig. S3, C and D), and the fluorescence of FLO6*^jap^*_dIDR2_ hardly recovered after photobleaching (fig. S2, D and E). This severe lack of recovery indicates that FLO6*^jap^*_dIDR2_ undergoes extensive aggregation, losing its liquid-like properties. These results suggest that IDR2 is crucial for preventing complete aggregation and maintaining the dynamic mobility of FLO6*^jap^* condensates.

We next examined whether IDR2 is important for starch synthesis in rice grains. Two types of transgenic plants expressing *FLO6^jap^* variants driven by the maize ubiquitin 1 promoter in *FLO6^jap^-KO1* background were generated. They were full-length *FLO6^jap^* fused to the GFP tag (named *Ubip:FLO6^jap^-GFPcomp*), and *FLO6^jap^* without IDR2 fused to the GFP tag (named *Ubip:FLO6^jap^_dIDR2_-GFPcomp*). Grains from *Ubip:FLO6^jap^-GFPcomp* homozygous plants were translucent with a high level of FLO6*^jap^*-GFP protein (Fig. 3A). The starch granule appearance and starch and amylose content of the translucent *Ubip:FLO6^jap^-GFPcomp* grains were the same as NPB grains (Fig. 3, B to D). However, expression of *FLO6^jap^_dIDR2_-GFP* under the control of maize ubiquitin 1 promoter failed to restore the translucent phenotype in *FLO6^jap^-KO1* grains, likely due to the notably lower FLO6*^jap^*_dIDR2_-GFP protein level compared to FLO6*^jap^* in NPB (Fig. 3A).

**Fig. 3.**
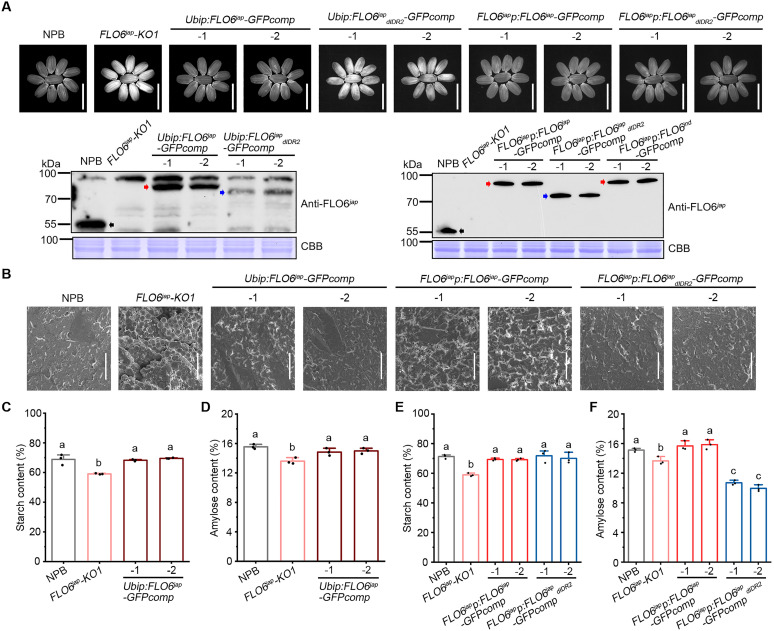
The second IDR (IDR2) of FLO6*^jap^* affects amylose synthesis in rice grain. (**A**) Representative grains from NPB, *FLO6^jap^-KO1*, and *FLO6^jap^-KO1* complemented plants by overexpressing *FLO6^jap^-GFP* or *FLO6^jap^_dIDR2_-GFP*, driven by the maize ubiquitin 1 promoter (Ubip) or authentic *FLO6^jap^* promoter. Scale bars, 1 cm. Protein levels of FLO6 (black arrow), FLO6-GFP (red arrow), and FLO6*^jap^*_dIDR2_-GFP (blue arrow) in the 15-DAP endosperm of the indicated plants were determined by immunoblotting. Coomassie blue staining was used to check for equal loading of samples. (**B**) SEM analyses of the endosperm of NPB and *FLO6^jap^-KO1* and *FLO6^jap^-KO1* complemented grains. Scale bars, 50 μm. (**C** to **F**) Starch and amylose content of NPB and *FLO6^jap^-KO1* and *Ubip:FLO6^jap^-GFPcomp* [(C) and (D)], and *FLO6^jap^p:FLO6^jap^-GFPcomp* and *FLO6^jap^p:FLO6^jap^_dIDR2_-GFPcomp* [(E) and (F)] grains. Values are means ± SD (*n* = 3). Different letters in (C) to (F) indicate significant differences (*P* < 0.05) based on the one-way analysis of variance (ANOVA) analysis followed by the pairwise post hoc Tukey test.

To further investigate the function of IDR2 of FLO6*^jap^*, we generated transgenic plants expressing *FLO6^jap^_dIDR2_-GFP* driven by the native *FLO6^jap^* promoter in the *FLO6^jap^-KO1* background (designated as *FLO6^jap^p:FLO6^jap^_dIDR2_-GFPcomp*). The protein levels of FLO6*^jap^*_dIDR2_-GFP in *FLO6^jap^p:FLO6^jap^_dIDR2_-GFPcomp* developing grains were similar to that of *FLO6^jap^p:FLO6^jap^-GFPcomp* and NPB grains (Fig. 3A). Moreover, grains from *FLO6^jap^p:FLO6^jap^_dIDR2_-GFPcomp* lines were translucent, and the microstructure of starch granules of *FLO6^jap^p:FLO6^jap^_dIDR2_-GFPcomp* was similar to that of NPB (Fig. 3, A and B). FLO6*^jap^*_dIDR2_-GFP also forms punctate condensates around starch granules in the 15-DAP (days after pollination) endosperm of *FLO6^jap^p:FLO6^jap^_dIDR2_-GFPcomp* grains, compared to FLO6*^jap^*-GFP in *FLO6^jap^p:FLO6^jap^-GFPcomp* grain (Fig. 2B). FRAP assays demonstrated that the recovery of fluorescence of the FLO6*^jap^*_dIDR2_-GFP condensates in *FLO6^jap^p:FLO6^jap^_dIDR2_-GFPcomp* after photobleaching was much slower and substantially lower in comparison to the FLO6*^jap^*-GFP in *FLO6^jap^p:FLO6^jap^-GFPcomp* lines (Fig. 2C), further supporting that the loss of IDR2 promotes aggregation in vivo. Besides, condensates of FLO6*^jap^*_dIDR2_ can also recruit GBSS1^b^ in vitro (Fig. 2E). These results indicate that IDR2 of FLO6*^jap^* is crucial for FLO6 phase separation in vitro and in vivo.

### An SNP determines differential condensation of FLO6 alleles between *japonica* and *indica* rice subspecies

Generally, there is a large difference in grain amylose content between *japonica* and *indica* rice cultivars. In view of the importance of FLO6*^jap^* in amylose synthesis of *japonica* rice cultivar NPB, we assumed that the *FLO6* allele in *indica* rice cultivars might be also crucial for grain amylose synthesis. Sequence alignment reveals that there is only one amino acid change (from Gly^382^ to Arg^382^) in FLO6 between NPB (*O. sativa* L. ssp. *japonica*) and 93-11 (*O. sativa* L. ssp. *indica*) due to a base change from guanine^3194^ to adenine (we named this base pair change SNP^3194^), which is within the IDR2 (fig. S4A). Notably, Gly^382^ is just between two α helices according to the protein structure prediction of FLO6*^jap^* (fig. S4B), which might result in a different structural flexibility of FLO6 alleles due to the different electric charge between Gly^382^ and Arg^382^.

To validate the phase separation property of FLO6 from *indica* rice, FLO6*^ind^* and FLO6*^ind^*-GFP were expressed and purified in vitro (fig. S2A). FLO6*^ind^* and FLO6*^ind^*-GFP proteins formed amorphous gel-like condensates (fig. S2B). FRAP assays on FLO6*^ind^*-GFP and FLO6*^ind^* condensates showed that the recovery ability of FLO6*^ind^* was decreased compared to FLO6*^jap^* and recovered to a plateau of only about 40% of the fluorescence intensity after bleaching (fig. S2, D and E). This lower recovery plateau indicates a larger immobile fraction, suggesting that FLO6*^ind^* condensates are more prone to aggregation than FLO6*^jap^*. Moreover, the formation of FLO6*^ind^*-GFP condensate was also prevented by 7.5% (m/V) 1,6-hexanediol as also for FLO6*^jap^*-GFP (fig. S2H).

To further validate that SNP^3194^ of *FLO6* affects the properties of FLO6*^ind^* condensates in developing grains, we created *FLO6^jap^p::FLO6^ind^-GFP* transgenic plants in the *FLO6^jap^-KO1* background (designated as *FLO6^jap^p:FLO6^ind^-GFPcomp*). Grains from *FLO6^jap^p:FLO6^ind^-GFPcomp* plants were translucent and accumulated similar levels of FLO6*^ind^*-GFP compared to FLO6*^jap^*-GFP in *FLO6^jap^p:FLO6^jap^-GFPcomp* and FLO6*^jap^* in NPB grains (Figs. 3A and 4A). The microstructure of starch granules of *FLO6^jap^p:FLO6^ind^-GFPcomp* grains was restored to that of *FLO6^jap^p:FLO6^jap^-GFPcomp* and NPB (Fig. 4B).

**Fig. 4.**
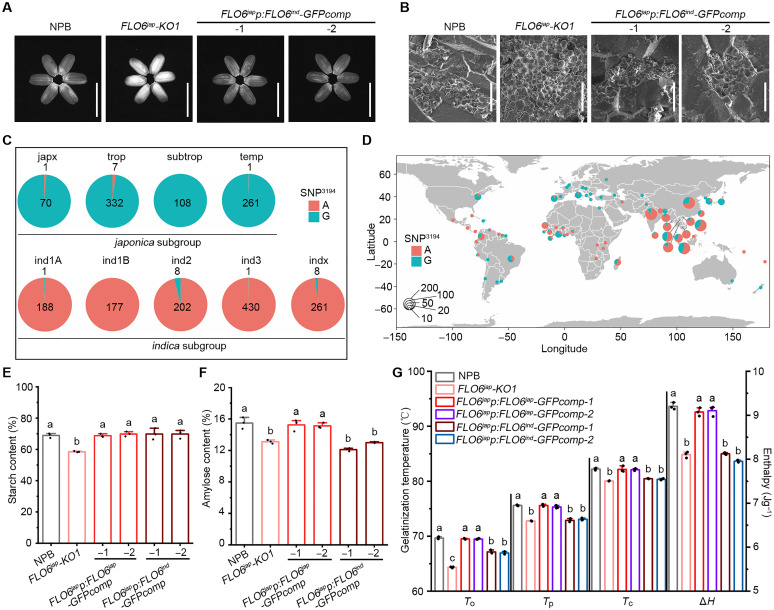
An SNP in IDR2 of *FLO6* alleles is crucial for amylose synthesis in rice grain. (**A**) Representative grains from NPB, *FLO6^jap^-KO1*, and *FLO6^jap^-KO1* complemented plants by overexpressing *FLO6^ind^-GFP* (named *FLO6^jap^p:FLO6^ind^-GFPcomp*, driven by the authentic *FLO6^jap^* promoter). Scale bars, 1 cm. (**B**) SEM analyses of the endosperm of NPB and *FLO6^jap^-KO1* and *FLO6^jap^p:FLO6^ind^-GFPcomp* grains. Scale bars, 50 μm. (**C** and **D**) Subpopulation and geographic distribution of SNP^3194^ of *FLO6* in 2277 rice accessions. Numbers in (C) indicate the accession count with adenine or guanine genotype. (**E** and **F**) Starch and amylose content of NPB and *FLO6^jap^-KO1* and *FLO6^jap^p:FLO6^ind^-GFPcomp* grains. Values are means ± SD (*n* = 3). (**G**) Thermal gelatinization temperature of endosperm starch of NPB and *FLO6^jap^-KO1* and *FLO6^jap^-KO1* complemented grains. *T*_o_, *T*_p_, *T*_c_, and Δ*H* represent the onset, peak, and conclusion gelatinization temperatures and gelatinization enthalpy, respectively. Values are means ± SD (*n* = 3). Different letters in (E) to (G) indicate significant differences (*P* < 0.05) based on the one-way ANOVA analysis followed by the pairwise post hoc Tukey test.

We also examined the FLO6*^ind^*-GFP condensates in *FLO6^jap^p:FLO6^ind^-GFPcomp* grains by fluorescence microscopy. The results revealed that FLO6*^ind^*-GFP condensates were also mostly distributed around starch granules, and the area of some FLO6*^ind^*-GFP condensates was slightly larger than that of FLO6*^jap^*-GFP in 15-DAP endosperm of *FLO6^jap^p:FLO6^jap^-GFPcomp* grains (Fig. 2B). FRAP assays demonstrated that the fluorescence of FLO6*^ind^*-GFP condensates recovered to roughly 50% of its prebleaching intensity after the bleaching event, which is lower than for FLO6*^jap^*-GFP droplets in 15-DAP endosperm of *FLO6^jap^p:FLO6^jap^-GFPcomp* grains (Fig. 2C), consistent with the in vitro observation of increased aggregation behavior. These results indicate that the natural variation at the SNP^3194^ locus affects the mobility and aggregation propensity of FLO6 condensates in different rice subspecies, which may be associated with the regulation on GBSS1 activity.

### Base editing on SNP^3194^ of *FLO6^jap^* leads to dramatic changes of rice agronomic traits

Overall, there are nine SNPs located within the exons of the *FLO6* gene in rice populations, and only SNP^3194^ (named vg0327411523 in database) exhibits obviously divergent distribution between the analyzed *japonica* and *indica* samples, according to the data from RiceVarMap2 database (fig. S4C). Considering the importance of SNP^3194^, we next analyzed the variation of SNP^3194^ in *japonica* and *indica* species with the 3000 rice genomes (3K-RG) data (table S2) (*19*). Almost all the *japonica* accessions have guanine, whereas most *indica* accessions have adenine (Fig. 4C). Geographic distribution of SNP^3194^ showed that guanine is widely distributed in temperate regions, while adenine mainly exists in tropical and subtropical areas (Fig. 4D), indicating a relation between condensation of FLO6 alleles and geographical and thermal adaptation of the different rice subspecies.

The distribution of SNP^3194^ between *japonica* and *indica* rice cultivars prompted us to examine whether alteration of SNP^3194^ is an efficient way to change amylose content in rice grains. Although the starch content of *FLO6^jap^p:FLO6^ind^-GFPcomp* grains was restored to the level in NPB (Fig. 4E), the amylose content of the transgenic grains was dramatically decreased to about 13% (Fig. 4F). The lower amylose content in *FLO6^jap^p:FLO6^ind^-GFPcomp* grains prompted us to measure the gelatinization properties of endosperm starch. The results showed that the onset (*T*_o_), peak (*T*_p_), and conclusion (*T*_c_) temperatures as well as the gelatinization enthalpy (Δ*H*) of endosperm starch in *FLO6^jap^-KO1* and *FLO6^jap^p:FLO6^ind^-GFPcomp* grains were all strikingly reduced compared to those of NPB grains and *FLO6^jap^p:FLO6^jap^-GFPcomp* (Fig. 4G). Together, these data support the conclusion that a single amino acid alteration changes FLO6 condensation behavior and is associated with altered amylose synthesis in *japonica* and *indica* rice subspecies.

To rule out the possibility that changes in amylose content are caused by the fusion of the GFP tag to FLO6 alleles, we generated base-edited plants of *FLO6^jap^* with an adenine in NPB. We obtained two mutant lines with the guanine-to-adenine conversion at base 3194 in *FLO6^jap^*, and we named them *FLO6^jap-3194(A/G)^-9* and *-25* (fig. S4D). We next screened progeny seedlings from *FLO6^jap-3194(A/G)^* in the second (T1) and third (T2) generation and selected transgene-free (Cas9-free) homozygous lines of *FLO6^jap-3194(A/A)^* and *FLO6^jap-3194(G/G)^* (figs. S4E and S5). To rigorously evaluate potential unintended genome alterations and rule out off-target effects, we performed whole-genome sequencing (WGS) on these selected T2 lines (fig. S5B). Our results demonstrated no detectable off-target mutations in the homozygous edited *FLO6^jap-3194(A/A)^* lines (fig. S5C and table S3) (*20*).

The shape of selected *FLO6^jap-3194(A/A)^* and *FLO6^jap-3194(G/G)^* mature grains was similar to that of NPB (Fig. 5A). We firstly analyzed starch characteristics of mature grains from *FLO6^jap-3194(A/A)^* and *FLO6^jap-3194(G/G)^* in the T2 generation. The appearance of starch granules and starch content from *FLO6^jap-3194(A/A)^* and *FLO6^jap-3194(G/G)^* grains were very similar as in NPB (Fig. 5, B and C). However, the amylose content of *FLO6^jap-3194(A/A)^* grains was notably lower than that of *FLO6^jap-3194(G/G)^* and NPB (Fig. 5D). Next, we evaluated the gelatinization properties of endosperm starch in *FLO6^jap-3194(A/A)^* grains. *T*_o_, *T*_p_, and *T*_c_ as well as the Δ*H* of gelatinization of endosperm starch in *FLO6^jap-3194(A/A)^* grains were all substantially lower than those of *FLO6^jap-3194(G/G)^* and NPB grains (Fig. 5E), which suggests that the eating and cooking quality has been changed in *FLO6^jap-3194(A/A)^* grains. Moreover, base editing of SNP^3194^ in *FLO6^jap^* had barely any negative impact on the grain yield of the edited plants (Fig. 5, F and G).

**Fig. 5.**
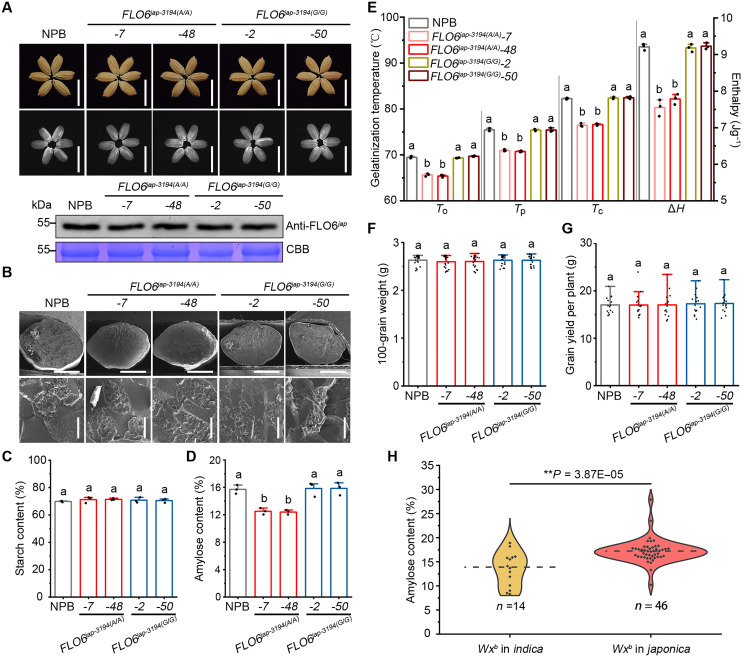
Base editing of guanine^3194^ of *FLO6^jap^* to adenine alters amylose content of NPB grain. (**A**) Representative raw and dehulled grains from NPB, and edited T2 (third generation) *FLO6^jap-3194(A/A)^* and *FLO6^jap-3194(G/G)^* plants. Scale bars, 1 cm. The protein levels of FLO6*^ind^* (edited FLO6*^jap^*) or FLO6*^jap^* in the 15-DAP endosperm of the indicated plants as determined by immunoblotting are shown. Coomassie blue staining was used to check for equal loading of samples. (**B**) SEM analyses of the endosperm of NPB and edited T2 *FLO6^jap-3194(A/A)^* and *FLO6^jap-3194(G/G)^* grains. Scale bars, 1 mm (top); 50 μm (bottom). (**C** and **D**) Starch and amylose content of NPB and edited T2 *FLO6^jap-3194(A/A)^* and *FLO6^jap-3194(G/G)^* grains. Values are means ± SD (*n* = 3). (**E**) Thermal gelatinization temperature of endosperm starch of NPB and edited T2 *FLO6^jap-3194(A/A)^* and *FLO6^jap-3194(G/G)^* grains. *T*_o_, *T*_p_, *T*_c_, and Δ*H* represent the onset, peak, and conclusion gelatinization temperatures and gelatinization enthalpy, respectively. Values are means ± SD (*n* = 3). (**F** and **G**) One hundred–grain weight and grain yield per plant of NPB and edited T2 *FLO6^jap-3194(A/A)^* and *FLO6^jap-3194(G/G)^*. Values are means ± SD [*n* = 20 in (F), *n* = 15 in (G)]. (**H**) Amylose content of grains of 46 *japonica* accessions with *Wx^b^* and 14 *indica* accessions with *Wx^b^*. The median is shown by the dashed line. The asterisks indicate statistical significance determined by a Student’s *t* test (**P < 0.01). Different letters in (C) to (G) indicate significant differences (*P* < 0.05) based on the one-way ANOVA analysis followed by the pairwise post hoc Tukey test.

To further assess whether the FLO6-mediated regulation extends beyond our transgenic system to natural populations, we evaluated several accessions from different rice subspecies with reported *Wx^b^* haplotype and amylose content (table S4) (*6*, *21*, *22*). The statistics showed that *japonica* accessions with *Wx^b^* generally had much higher amylose content than *indica* accessions with *Wx^b^* (Fig. 5H). These results suggest that rice subspecies harboring FLO6*^jap^* may produce higher levels of amylose via GBSS1^b^ compared to those carrying FLO6*^ind^*. Together, these results indicate that SNP^3194^ of *FLO6* alleles is a promising target for specifically decreasing amylose content in *japonica* rice cultivars.

### FLO6 alleles differentially regulate GBSS1^b^ activity in rice grain

To determine whether the low amylose content in *FLO6^jap-3194(A/A)^* grains is attributed to reduced GBSS1^b^ activity, relative to that in NPB and *FLO6^jap-3194(G/G)^* grains, we sequentially measured the amount and activity of GBSS1^b^ in 15-DAP endosperm of *FLO6^jap-3194(A/A)^* and *FLO6^jap-3194(G/G)^* grains, where the expressions of both *FLO6* alleles are regulated by the *FLO6^jap^* native promoter. Results showed that GBSS1^b^ activity in endosperm from two independent *FLO6^jap-3194(A/A)^* lines was notably lower than in two independent *FLO6^jap-3194(G/G)^* lines and NPB (Fig. 6A), in spite of the similar amount of GBSS1^b^ and FLO6 alleles in 15-DAP endosperm of the analyzed plants (Fig. 6, A and B).

**Fig. 6.**
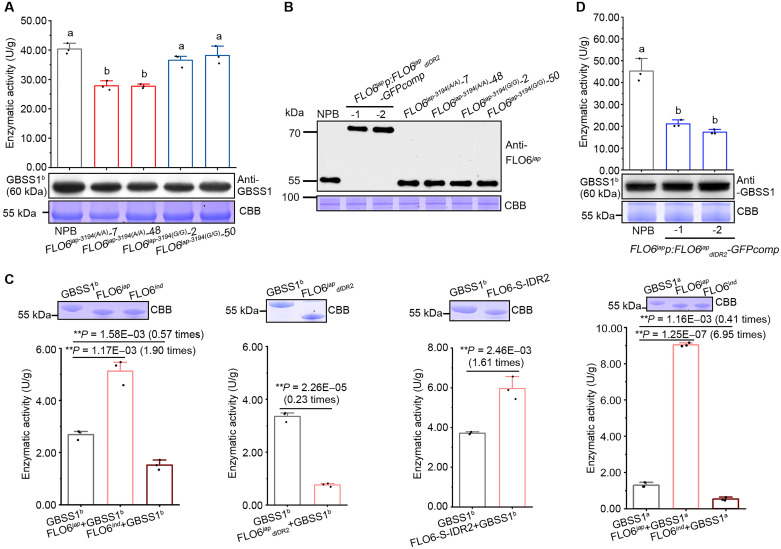
FLO6 alleles regulate GBSS1^b^ activity differently leading tosubstantial differences in amylose content in rice grains. (**A** and **D**) GBSS1^b^ amount and activity in the 15-DAP endosperm from NPB and *FLO6^jap-3194(G/G)^* and *FLO6^jap-3194(A/A)^* (A), and *FLO6^jap^p:FLO6^jap^_dIDR2_-GFPcomp* (D) grains. Protein levels of GBSS1^b^ in the 15-DAP endosperm of indicated plants were determined by immunoblotting. Coomassie blue staining was used to check for equal loading of samples. Values are means ± SD (*n* = 3). (**B**) Protein levels of FLO6*^jap^*_dIDR2_-GFP and FLO6*^jap^* and FLO6*^ind^* (edited FLO6*^jap^*) in the 15-DAP endosperm of the indicated plants were determined by immunoblotting. Coomassie blue staining was used to check for equal loading of samples. (**C**) Activity measurements of purified GBSS1^b^ with addition of purified FLO6 and variants. Values are means ± SD (*n* = 3); activity fold changes of GBSS1 with added FLO6 and variants compared to GBSS1 are shown. Coomassie blue staining was used for quantification of the purified proteins. Asterisks indicate statistical significance determined by a Student’s *t* test (**P < 0.01). Different letters in (A) and (D) indicate significant differences (*P* < 0.05) based on the one-way ANOVA analysis followed by the pairwise post hoc Tukey test.

As condensates of FLO6 were able to recruit GBSS1^b^ in vitro and in vivo (Fig. 2, E and F), we next examined whether GBSS1^b^ activity is differentially regulated by FLO6 condensates. To test our hypothesis, we incubated purified GBSS1^b^ with three types of FLO6 condensates (FLO6*^jap^*, FLO6*^ind^*, and FLO6*^jap^*_dIDR2_) with different mobility, respectively, and measured the activity of GBSS1^b^ after incubation (fig. S6A). The results showed that the activity of GBSS1^b^ was dramatically increased by addition of FLO6*^jap^* condensates, but remarkably decreased by addition of FLO6*^ind^* condensates, compared to the control group with only GBSS1^b^ (Fig. 6C). As expected, incubation of FLO6*^jap^*_dIDR2_ with GBSS1^b^ resulted in the lowest GBSS1^b^ activity (Fig. 6C), which is consistent with the fact that the FLO6*^jap^*_dIDR2_ condensates were more solid-like compared to the FLO6*^jap^* or FLO6*^ind^* condensates.

To substantiate the hypothesis that the mobility of FLO6 governs the activity of GBSS1^b^ and consequently influences amylose synthesis in rice grains, we also examined the regulatory effect of FLO6*^jap^*_dIDR2_ on GBSS1^b^ activity in vivo. The *FLO6^jap^p:FLO6^jap^_dIDR2_-GFPcomp* had the lowest GBSS1^b^ activity in developing endosperm, although it accumulated similar amounts of GBSS1^b^ and FLO6*^jap^*_dIDR2_-GFP (compared to FLO6), which correlates with the lowest amylose content in the grains, in comparison to NPB, *FLO6^jap-3194(A/A)^*, and *FLO6^jap-3194(G/G)^* (Figs. 3F, 4F, and 5D).

To definitively address whether the physical property of phase separation—rather than the specific primary sequence of IDR2—is responsible for regulating GBSS1^b^ activity, we constructed a FLO6*^jap^* chimera by replacing its native IDR2 (residues 375 to 418) with a heterologous sequence. To ensure functional compatibility, we screened published IDR-containing proteins capable of phase separation and identified a 44–amino acid fragment from yeast DNA topoisomerase 2 (*23*) (residues 1372 to 1415, named TOP2-F). This fragment closely matches not only the amino acid composition (e.g., proportions of charged, polar, and hydrophobic residues), but also the position-specific disorder confidence profile of the native IDR2 (exhibiting remarkably similar sequence-level disorder probabilities along the 44–amino acid stretch). The resulting chimeric protein, named FLO6-S-IDR2, retained phase separation behavior and readily formed liquid droplets in vitro (fig. S6, B to D). FRAP assays showed that the fluorescence recovery dynamics of FLO6-S-IDR2 condensates were comparable to those of FLO6*^jap^* (fig. S6E), indicating that the chimeric substitution successfully restored high condensate mobility. We then incubated purified GBSS1^b^ with FLO6-S-IDR2 and measured GBSS1^b^ activity. Notably, FLO6-S-IDR2 promoted GBSS1^b^ activity in vitro to a similar extent as FLO6*^jap^* condensates (Fig. 6C), indicating that LLPS is closely linked to FLO6 molecular function and likely drives the regulation of GBSS1 activity. However, our failure to obtain stable transgenic expression of the FLO6-S-IDR2 construct in rice plants suggests that the native IDR2 sequence context may also contribute to additional in vivo functions beyond phase separation, such as protein stability or proper plastid/amyloplast localization. Together, these findings suggest that the mobility of FLO6 condensates may contribute to the regulation of GBSS1 activity and thereby influence amylose synthesis in rice grains.

### Evolutionary origins and functional divergence of FLO6-GBSS1 allelic combinations

To trace the evolution of SNP^3194^ in *FLO6*, we examined the sequences of *FLO6* with the Basic Local Alignment Search Tool (BLAST) in a diverse sample of 366 wild rice including two *Oryza rufipogon* [Ruf1 (further divided into Ruf1a and Ruf1b) and Ruf2] and two *O. nivara* (Niv1 and Niv2) lineages reported previously (table S2) (*24*). Specifically, 91% of 46 Ruf1a (mainly in Southern China), 85% of 27 Ruf1b, 89% of 131 Ruf2 individuals, and nearly all the 87 Niv1 individuals contain guanine at SNP^3194^ (Fig. 7, A and B, and fig. S7). In contrast, 75% of 75 Niv2 individuals have adenine at SNP^3194^, suggesting that *O. sativa* L. ssp. *indica* likely inherited the adenine from Niv2, which is mainly distributed in South and Southeast Asia (Fig. 7, A and B). The distribution pattern of SNP^3194^ further demonstrates that this polymorphism in cultivated rice is strictly partitioned between *japonica* and *indica* subspecies, reflecting their distinct genetic divergence inherited from different wild ancestors (Figs. 4C and 7A).

**Fig. 7.**
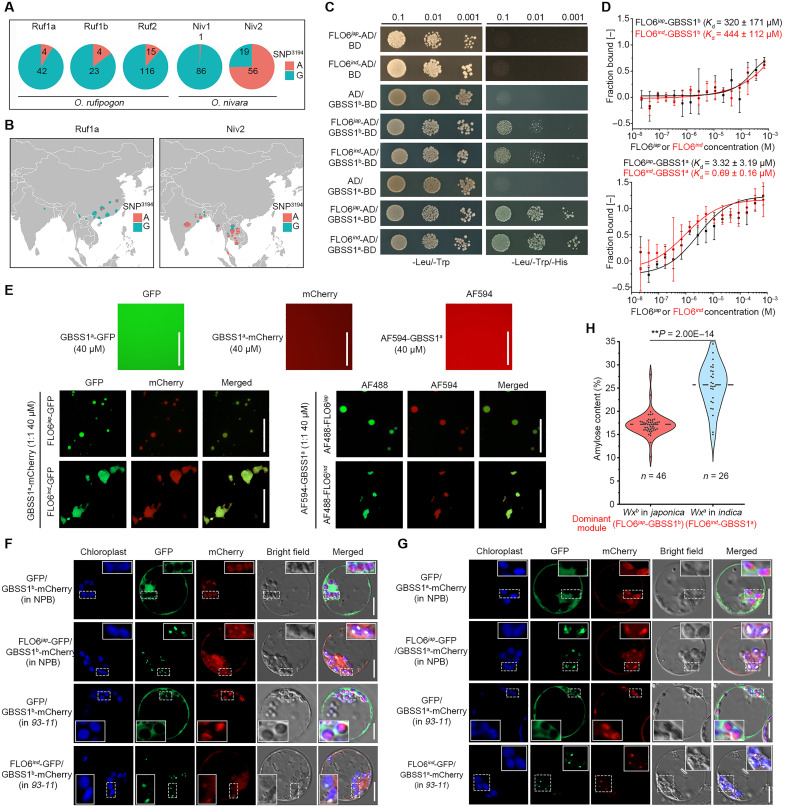
Analysis of the evolution of the FLO6-GBSS1 module in rice. (**A**) Subpopulation distribution of SNP^3194^ of *FLO6* in 366 wild rice individuals. Numbers indicate the count of rice individuals with adenine or guanine genotype. (**B**) Geographical distribution of SNP^3194^ of *FLO6* in Ruf1a and Niv2. (**C** and **D**) Yeast two-hybrid and MST assays were used to assess interactions between FLO6 alleles and GBSS1 alleles. Values are means ± SD (*n* = 3) in (D). (**E**) Phase separation of GBSS1^a^ labeled with different fluorescent tags and dyes, and in vitro recruitment assays of purified GFP-tagged FLO6 alleles with GBSS1^a^-mCherry, or AF488-labeled FLO6 alleles with AF594-GBSS1^a^. For recruitment, GFP-tagged FLO6 variants (retaining the 6 × His tag) were incubated with GBSS1^a^-mCherry, or AF488-labeled FLO6 variants were incubated with AF594-GBSS1^a^. The 6 × His tags were cleaved from all GBSS1^a^ proteins and dye-labeled FLO6 proteins, while GFP-tagged FLO6 variants retained the 6 × His tag. All proteins were used at 40 μM. Scale bars, 50 μm. (**F** and **G**) Fluorescence microscopy of NPB and *93-11* mesophyll protoplasts expressing FLO6*^jap^*-GFP or FLO6*^ind^*-GFP in combination with GBSS1^b^-mCherry and GBSS1^a^-mCherry under continuous dark conditions individually. Scale bars, 10 μm. Representative colocalization regions are marked by white boxes; enlargements of the colocalization regions are displayed in the corner of pictures. (**H**) Amylose content of grains of 46 *japonica* accessions with *Wx^b^* and 26 *indica* accessions with *Wx^a^*. The median is showed by the dashed line. Asterisks indicate statistical significance determined by a Student’s *t* test (**P < 0.01).

To explore the functional implications of this evolutionary partition, we next evaluated how these FLO6 alleles interact with the predominant GBSS1 variants found in *indica* (GBSS1^a^) and *japonica* (GBSS1^b^) cultivars. Notably, both yeast two-hybrid and MST assays showed that the strength of interaction between FLO6 alleles and GBSS1^a^ is much stronger than that between FLO6 alleles and GBSS1^b^ (Fig. 7, C and D, and fig. S8A). Besides, condensates of FLO6 alleles both recruited GBSS1^b^ and GBSS1^a^ readily in vitro and in vivo (Figs. 2, E and F, and 7, E to G, and fig. S8A). Unexpectedly, in vitro assays of GBSS1 activity showed that FLO6*^jap^* enhanced GBSS1^a^ activity much more compared to GBSS1^b^, while FLO6*^ind^* decreased the activity of GBSS1^a^ more than that of GBSS1^b^ (Fig. 6C). Our in vitro assays do not necessarily reflect the actual GBSS1 activity within the complex starch granule matrix in vivo. Nevertheless, these in vitro observations provide a simplified system that clearly unveils the differential biophysical regulatory potential of FLO6 variants on distinct GBSS1 alleles.

To validate the biological relevance of these molecular observations at the population level, we surveyed the amylose content of natural rice accessions carrying the predominant FLO6-GBSS1 allelic combinations. The analysis revealed that *indica* accessions with the highly expressed *Wx^a^* allele exhibit substantially higher amylose content than *japonica* accessions with the *Wx^b^* allele (Fig. 7H). This macroscopic pattern is fully consistent with the established role of *Wx* allelic variation in determining baseline GBSS1 protein abundance. However, when considered together with our finding that *japonica* accessions carrying *Wx^b^* still have higher amylose than *indica* accessions with *Wx^b^* (Fig. 5H), these data collectively demonstrate that the *Wx*-determined enzyme baseline and the FLO6-mediated biophysical fine-tuning of GBSSl activity collectively shape the amylose landscape across rice subspecies.

### Structural basis and evolutionary conservation of the FLO6-GBSS1 interface

To elucidate the structural mechanism underlying the distinct interactions and diverse regulatory effects of these FLO6-GBSS1 allelic combinations, we used AlphaFold 3 to model the protein complex (fig. S4B). The predicted complex indicates that the major interface involves the long helix α5 of FLO6 and helix α10 of GBSS1. Furthermore, structural analysis provides insight into how the single amino acid substitution between the two alleles (Ser^415^ in GBSS1^a^ versus Pro^415^ in GBSS1^b^) affects this interaction. Specifically, Pro^415^ in GBSS1^b^ localizes to the C-terminal end of helix α11, where it stabilizes the local hydrophobic core. This local stabilization may constrain the surrounding tertiary structure, indirectly reducing the accessibility of the binding surface to FLO6. Conversely, Ser^415^ in GBSS1^a^ likely weakens this hydrophobic packing and increases conformational flexibility, favoring a broader interaction interface with FLO6. In addition, the predicted model revealed that the key IDR2 of FLO6 (residues 375 to 418) forms a helix-turn-helix-like region (α4-loop-α5) that contributes directly to GBSS1 binding, specifically through residues 408 to 418 (fig. S4B).

Considering that variation at FLO6 residue 382 and GBSS1 residue 415 may contribute to the diverse functions of FLO6-GBSS1 modules, we further evaluated sequence conservation across rice and more distantly related *Gramineous* plants. Consistent with the structural model, amino acid sequence alignments revealed that the interface residues responsible for GBSS1 recognition (FLO6 residues 408 to 418) are highly conserved, whereas the adjacent loop region (residues 375 to 389, containing the polymorphic residue 382) exhibits relatively low conservation (fig. S8B). Similarly, residue 415 of GBSS1 and its overall sequence exhibit high conservation across species, which is crucial for the basic catalytic activity of GBSS1 homologs (fig. S8B). These results suggest that while the physical interface and basic activity are evolutionarily stable, variations in the less conserved regions (e.g., FLO6 residue 382) likely modulate the properties of FLO6 phase separation during the evolution of *Gramineous* plants, thereby providing additional means to precisely regulate GBSS1 activity in different genetic backgrounds (fig. S8B). Collectively, these studies demonstrate that while the allelic variation of GBSS1 itself is the primary determinant of amylose content (driven by splicing efficiency and protein abundance), the natural variation of *FLO6*, which likely coevolved with *Wx*, acts as an additional evolutionary layer to fine-tune GBSS1 activity in different genetic backgrounds.

## DISCUSSION

Amylose content is a key factor determining the quality of rice starch, yet the regulatory mechanisms underlying its variation among different rice subspecies remain unclear (*5*, *25*, *26*). In this study, we demonstrate that FLO6 undergoes phase separation to form condensates that adhere to starch granule surfaces, recruit GBSS1, and differentially regulate its activity during amylose synthesis. A single SNP (SNP^3194^) in the IDR2 of FLO6, strictly partitioned between *japonica* and *indica* subspecies, determines the mobility of FLO6 condensates and thereby fine-tunes amylose content without affecting starch content or grain yield (Fig. 5, C to G). These findings identify SNP^3194^ as a promising target for directionally lowering grain amylose levels in traditional *japonica* cultivars.

To understand how this single SNP exerts such specific control over amylose without disrupting overall granule morphology, it is essential to consider the spatial organization of starch synthesis. The growth of starch granules relies on the coordinated synthesis of amylose and amylopectin (*1*). Amylopectin expands the granule through its branched chains stacked in ordered crystalline layers (historically described by the “surface growth model”) (*27*). Classically, amylose synthesis is thought to occur predominantly after GBSS1 is buried within this semicrystalline matrix, protecting nascent amylose from degradation. However, our in planta imaging reveals that FLO6 and GBSS1 colocalize at distinct sites on the granule surface, whereas without FLO6, GBSS1 is diffusely distributed (Fig. 2F).

To reconcile our observations with established biochemical view, we propose a “Surface Recruitment and Progressive Burial” model. Through its CBM domain, phase-separated FLO6 anchors to the starch surface and acts as a specialized “loading hub” to recruit the CBM-lacking GBSS1. Our results indicate that this accurate spatial positioning is a prerequisite for normal amylose synthesis and the formation of transparent grains (Fig. 2F). As the amylopectin matrix continuously crystallizes and expands, these surface-anchored FLO6-GBSS1 condensates are progressively engulfed and buried into the alternating lamellae, where the bulk of protected amylose elongation occurs.

While confocal imaging typically captures only one or two large “macro-condensates” per granule (Fig. 2, B, D, and F), this likely reflects a spatial snapshot constrained by the physical sectioning required for endosperm observation. Given that starch granules are inherently dense, opaque three-dimensional (3D) structures, the detectable fluorescence is strictly limited to the specific 2D plane exposed by the microtome cut. Consequently, condensates distributed across other spatial layers are either physically excised during sample preparation or obscured by the opaque starch matrix. Beyond these physical constraints, the detection limits of conventional fluorescence microscopy mean we likely only resolve the largest, most highly coalesced droplets. It is therefore highly probable that numerous subdiffraction “microcondensates” simultaneously coat the entire granule surface to ensure uniform enzyme loading. Furthermore, considering that FLO6 undergoes dynamic transitions between punctate and ring-shaped signals in response to light/dark cycles (*14*), the protein may also adopt alternative physical states—such as continuous rings encircling the granules—depending on the environmental conditions or developmental stages.

A crucial point is that the severe morphological defects in *flo6* starch granules cannot be attributed simply to reduced amylose, which is nonessential for overall granule morphogenesis. Instead, FLO6 likely functions as a “multienzyme scaffold.” Beyond GBSS1, FLO6 interacts with key amylopectin-modifying enzymes like isoamylase 1, starch synthase IVb (SSIVb) (*14*, *17*), and the condensate-forming OsLESV, which codistributes with FLO6 and isoamylase 1 within the internal lamellae (*28*). Thus, FLO6 condensates serve as a multilayered platform coordinating both amylose and amylopectin synthesis. Based on this framework, natural variations in FLO6 condensate mobility (e.g., FLO6*^jap^* and FLO6*^ind^*) fine-tune GBSS1 recruitment and activity, thereby altering amylose content while maintaining normal overall granule morphology. In contrast, the complete absence of FLO6 condensates in *FLO6-KO1* triggers the collapse of this multienzyme scaffold. The resulting diffuse distribution of vital enzymes (Fig. 2F) uncouples localized starch synthesis and modification, ultimately leading to random nucleation, unordered crystallization, and severely deformed starch granules (Fig. 8).

**Fig. 8.**
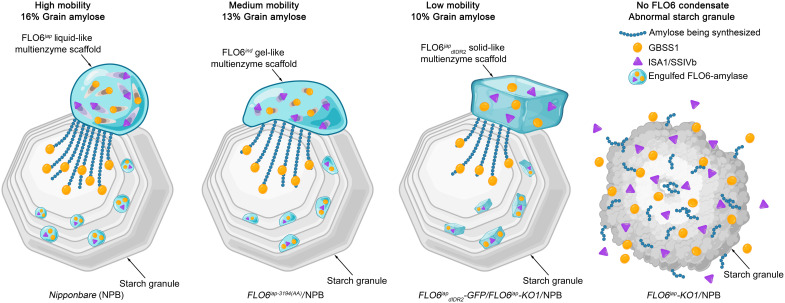
A “Surface Recruitment and Progressive Burial” model for the multilayered regulation of starch biosynthesis by the FLO6 scaffold. Schematic illustration of how the physical state of FLO6 condensates coordinates enzyme recruitment and starch granule morphogenesis. Left three panels: In wild-type and complemented lines, phase-separated FLO6 (cyan) acts as a “multienzyme scaffold” on the granule surface, recruiting GBSS1 (orange spheres) and amylopectin-modifying enzymes such as ISA1/SSIVb (purple tetrahedrons). This spatial organization serves as a frontline “loading hub.” As the starch matrix (light gray) expands through surface growth, these surface-anchored complexes are progressively engulfed and buried into the alternating lamellae. Bulk amylose elongation (dark blue threads) occurs primarily after the recruitment of GBSS1 into the protective granular matrix. The mobility of the scaffold determines enzymatic efficiency: Highly dynamic liquid-like FLO6*^jap^* condensates exhibit the highest GBSS1 activity, while the more viscous FLO6*^ind^* (gel-like) and rigid FLO6*^jap^*_dIDR2_ (solid-like) scaffolds lead to a progressive decrease in recruited enzyme activity and resulting amylose content. Although only a few macroscopic condensates are typically captured in 2D snapshots, they likely represent a cross-section of numerous subdiffraction microcondensates coating the 3D granule surface. Right: In the *flo6* mutant, the multienzyme scaffold collapses. The loss of coordinated recruitment leads to a diffuse distribution of starch biosynthetic enzymes (orange and purple) across the uneven surface. This absence of spatial control results in random nucleation, unordered crystallization, and disordered amylose orientation, ultimately causing the formation of severely deformed, hollow, and disorganized starch granules. This figure was created in BioRender. M. Ouyang (2026); https://BioRender.com/tsgiht0.

Beyond orchestrating the spatial organization of starch-synthesizing enzymes, FLO6 condensates also directly influence the catalytic activity of the recruited GBSS1 (Fig. 6). The biophysical regulation mediated by *FLO6* must be contextualized within the dominant genetic impact of *Wx* alleles. It is well established that the splicing defect in the *japonica Wx^b^* allele drastically reduces mature GBSS1 protein levels compared to *indica* cultivars (*7*). To definitively isolate the function of FLO6 from this major background disparity, our in planta transgenic designs rigorously matched the protein levels of FLO6 variants within the identical NPB background (Figs. 3A, 5A, and 6B). This rigorous control demonstrates that the observed divergence in amylose synthesis is intrinsically driven by the SNP-induced phase properties of the FLO6 alleles, rather than differential protein abundance of the scaffold itself.

How do these distinct biophysical properties of FLO6 cooperate with varying GBSS1 levels in natural populations? We propose a “scaffold-client” stoichiometry model of biomolecular condensates. While phase separation generally enhances enzymatic reactions by concentrating substrates and catalysts (*29*), an imbalance in scaffold-client stoichiometry, driven by oversaturation or excessive intermolecular affinities, can trigger a transition from a liquid-like state to a percolated gel (*30*). Such a transition can attenuate molecular transport and suppress the activity of recruited proteins. Our germplasm survey suggests that the natural coselection of specific *FLO6* and *Wx* alleles has yielded optimized, lineage-specific amylose baselines (Figs. 5H and 7H).

In *japonica* cultivars carrying *Wx^b^*, the available GBSS1 client pool is limited. In this context, the highly mobile FLO6*^jap^* condensates act as efficient scaffolds to recruit and concentrate the scarce GBSS1 molecules, thereby serving as a biophysical “enhancer” that ensures sufficient amylose synthesis. Conversely, in *indica* cultivars carrying *Wx^a^*, the massive influx of highly abundant GBSS1^a^ clients dramatically alters the scaffold-client stoichiometry, driving the condensates toward gelation. Thus, the aggregation-prone nature of FLO6*^ind^* and its strong affinity for GBSS1^a^ act synergistically to suppress enzymatic hyperactivity. Here, FLO6*^ind^* serves as a sophisticated biophysical “buffer” against the massive accumulation of GBSS1^a^ in plastids.

This biophysical buffering hypothesis provides a compelling framework for both the evolutionary landscape and geographical adaptation of cultivated rice. Our germplasm analysis reveals that the specific genetic combination of the highly mobile FLO6*^jap^* scaffold with the highly expressed *indica Wx^a^* allele is extremely rare (table S4). We speculate that such a “hyperactive” configuration, lacking the necessary biophysical buffering, was strongly selected against during domestication to avoid detrimental extremes in grain physicochemical properties or plant fitness, thereby preserving the delicate balance required for optimal starch quality.

The evolutionary trajectory of SNP^3194^ provides profound genomic evidence for this coselection process (Fig. 7, A and B). Phylogenetic tracing indicates that the adenine variant can be traced back to wild *O. nivara* Niv2 populations predominantly distributed in tropical South and Southeast Asia, while guanine variant predominates in *O. rufipogon* and Niv1 lineages. Remarkably, this ancestral partition has been almost completely fixed during domestication: *japonica* accessions universally carry guanine, while *indica* accessions carry adenine (Fig. 4C). The strict co-inheritance of SNP^3194^ with the corresponding *Wx* alleles across distinct geographical zones (Figs. 4D and 7B) strongly suggests that the divergent condensation properties of FLO6 were coselected alongside *Wx* variation as a supplementary mechanism to optimize starch physicochemical properties for local adaptation. Considering that amylose content is crucial for seed development, dormancy, and germination across diverse growth environments (*31*–*33*), this module represents a refined evolutionary strategy for fine-tuning starch traits to fulfill local adaptation. Consequently, the strict intrasubspecies fixation of these alleles creates an inherent genetic bottleneck, making it fundamentally challenging to modulate amylose content through conventional *FLO6* allelic introgressions within the same subspecies.

Ultimately, our findings reveal that the FLO6-GBSS1 module coevolved as an essential, superimposed biophysical layer. By fine-tuning condensate mobility to match baseline enzyme abundance, this coordinated evolution optimally shapes the diverse amylose traits required for the global adaptation of modern rice subspecies.

## MATERIALS AND METHODS

### Plant materials and growth conditions

In this research, the coding regions (CDSs) of *FLO6^jap^* and *GBSS1^b^* were amplified by polymerase chain reaction (PCR) from cDNA of NPB (*O. sativa* L. ssp. *japonica*). *FLO6^jap^_dIDR2_* (with a deletion of the second intrinsically disordered region, IDR2) was generated by CDS fragments amplification and homologous recombination using the plasmid containing the *FLO6^jap^* CDS as the template. The coding sequence of *FLO6-S-IDR2*, containing the coding sequence of amino acids from the 1372nd to 1415th (DLSDSQPAIAPRSRASRAVAKKPTYVVDLSDDSFVDGDAEEDVE) of DNA topoisomerase 2 from *Eremothecium gossypii*, was obtained through gene synthesis by Sangon biotech. The CDS of *FLO6^ind^* was amplified by PCR from cDNA of *93-11* (*O. sativa* L. ssp. *indica*). GBSS1^a^, which differs from GBSS1^b^ at only one single amino acid (Ser^415^ in GBSS1^a^ versus Pro^415^ in GBSS1^b^). The CDS of GBSS1^a^ was generated by site-directed mutagenesis and amplification using the plasmid containing the GBSS1^b^ CDS as the template.

The *FLO6^jap^* KO mutants in the NPB background were generated using the *pBWA(V)HU-ylcas9*–based CRISPR-Cas9 system (hygromycin resistant). Briefly, two 20–nucleotide (nt) single guide RNAs (sgRNAs) (5′-CCTGACTACCCTCCCGAGGC-3′ and 5′-GAGGCGGAAGCTTCGCCGTC-3′) with specific recognition for *FLO6^jap^* were synthesized and annealed, and interlinked into the vector *pBWA(V)HU-ylcas9*, resulting in *pBWA(V)HU-ylcas9-FLO6^jap^*. The *GBSS1^b^* KO mutants in the NPB background were also generated using the *pBWA(V)HU-ylcas9*–based CRISPR-Cas9 system. Briefly, two 20-nt sgRNAs (5′-TCCTCGGTACGACCAGTACA-3′ and 5′-CCATTGCTACAAGCGTGGAG-3′) with specific recognition for *GBSS1^b^* were synthesized and annealed, and interlinked into the vector *pBWA(V)HU-ylcas9*, resulting in *pBWA(V)HU-ylcas9-GBSS1^b^*. The plasmid was transformed into NPB calli with *Agrobacterium* strain EHA105. *FLO6^jap^-KO* and *GBSS1^b^-KO* homozygous lines were identified by genome PCR and sequencing and phenotyping. Complementation of the *FLO6^jap^-KO1* mutant was achieved with plasmids constructed using a modified *pCAMBIA3300* vector [with the maize ubiquitin 1 (Ubi) promoter]. The coding sequences of *FLO6^jap^* fused to GFP tag (*FLO6^jap^-GFP*) and *FLO6^jap^* without IDR2 fused to GFP tag (*FLO6^jap^_dIDR2_-GFP*) were amplified and inserted downstream of the Ubi promoter in frame of the modified *pCAMBIA3300*, respectively, resulting in *Ubip:FLO6^jap^-GFP* and *Ubip:FLO6^jap^_dIDR2_-GFP* plasmids. These plasmids were transformed into *FLO6^jap^-KO1* homozygous calli with *Agrobacterium* strain EHA105.

Plasmids used for native promoter-driven *FLO6* and *GBSS1^b^* expression in rice were constructed using the *pCAMBIA3300* vector. The *FLO6^jap^* or *GBSS1^b^* native promoter sequences (2000 bp upstream of start codon ATG) from NPB and coding sequences of *FLO6^jap^-GFP*, *FLO6^jap^_dIDR2_-GFP*, *FLO6^ind^-GFP*, or *GBSS1^b^-mCherry* were amplified by PCR and recombined into *pCAMBIA3300* in combination, respectively, resulting in *FLO6^jap^p:FLO6^jap^-GFP*, *FLO6^jap^p:FLO6^jap^_dIDR2_-GFP*, *FLO6^jap^p:FLO6^ind^-GFP*, and *GBSS1^b^p:GBSS1^b^-mCherry*. The coding sequences of *GBSS1^b^p:GBSS1^b^-mCherry* was amplified by PCR and recombined into the *FLO6^jap^p:FLO6^jap^-GFP* plasmid in frame, resulting in *FLO6^jap^p:FLO6^jap^-GFP-GBSS1^b^p:GBSS1^b^-mCherry*. The plasmids *FLO6^jap^p:FLO6^jap^-GFP*, *FLO6^jap^p:FLO6^jap^_dIDR2_-GFP*, *FLO6^jap^p:FLO6^ind^-GFP*, *FLO6^jap^p:FLO6^jap^-GFP-GBSS1^b^p:GBSS1^b^-mCherry*, and *GBSS1^b^p:GBSS1^b^-mCherry* were transformed into *FLO6^jap^-KO1* homozygous calli with *Agrobacterium* strain EHA105, respectively.

To create point mutation of *FLO6^jap^* in NPB, we used the base editor *pH-ABE7-esgRNA* vector. The point mutation was introduced into the coding sequence of *FLO6^jap^* with annealed sgRNA oligo by Golden Gate. The resulting plasmid was transformed into NPB calli by *Agrobacterium* strain EHA105-mediated transformation. The sgRNA sequence is (5′-GACGGAGCAAAAGCACCAAA-3′). To identify the T0 rice edits containing the desired mutation among the transgenic plants, DNA was extracted from the leaf of edited rice individuals with an SDS-based DNA extraction method. Subsequently, PCR assays were carried out with the extracted DNA as template, and PCR products were next sequenced for identification of the target mutation. WGS of desired T2 edited rice plants was used to evaluate the off-target effect and was carried out by Majorbio company (Shanghai, China) according to the standard testing procedure. All the used primers are listed in table S5. For field experiments, all the wild-type NPB and mutants, and transgenic and base-edited plants were grown in a paddy field in Wuhan (118°E, 32°N) or Lingshui (110°E, 18°N) under local natural conditions.

### Protein expression and purification and fluorescence microscopy

Plasmids used for in vitro protein expression were constructed using pETMALc-H, pSmart-I, modified pET30-a-GFP, pSmart-I-GFP, and pSmart-I-mCherry vectors. All these vectors encode an N-terminal 6 × His tag to facilitate subsequent protein purification via Ni-NTA affinity chromatography. The coding sequences of *FLO6^jap^*, its variant *FLO6^jap^_dIDR2_*, *FLO6^ind^* and *FLO6-S-IDR2* without signal peptide (1 to 91 amino acids), and *GBSS1^b^* and *GBSS1^a^* without signal peptide (1 to 77 amino acids) were amplified by PCR and recombined into the expression vectors mentioned above, respectively, resulting in specific plasmids for in vitro protein expression. All the primers used are listed in table S5.

Plasmids for protein expression were transformed into BL21 (DE3) cells (CB105, Tiangen) through heat shock. *Escherichia coli* BL21 (DE3) cultures were grown at 37°C under constant shaking (180 rpm) in standard lysogeny broth medium. Before induction of protein expression, cultures were cooled down to 16°C. Proteins used in this study were expressed in *E. coli* BL21 (DE3) cells, with induction performed by adding 0.5 mM isopropyl β-d-1-thiogalactopyranoside and subsequent incubation at 16°C overnight. The cells were collected and resuspended in lysis buffer [50 mM tris (pH 7.4), 500 mM NaCl, 10% glycerol, and 1 mM phenylmethylsulfonyl fluoride] and then lysed using an ultrasonic homogenizer (JY92-IIDN, Scientz) and centrifuged. The supernatants were first purified with Ni-NTA Sefinose resin (Smart-Lifesciences) and subsequently dialyzed into a protein storage buffer consisting of 30 mM tris-HCl (pH 7.5) and 500 mM NaCl.

Subsequent purification steps varied depending on the specific expression vector. For proteins expressed from the modified pET30a-GFP vector, the 6 × His tag was retained without further cleavage. Conversely, for target proteins expressed from the pETMALc-H, pSmart-I, pSmart-I-GFP, and pSmart-I-mCherry vectors, the fusion tags (MBP-His or SUMO-His) were enzymatically removed following dialysis. This was achieved by incubating the dialyzed proteins with either TEV protease (Novoprotein, PE004) to remove the MBP tag, or SUMO protease (Novoprotein, PE007) to remove the SUMO tag. After cleavage, these specific target proteins were further polished using a Superdex 200 Increase 10/300 GL size exclusion chromatography column (SD200, GE Healthcare). The peak fractions containing the target proteins were pooled and concentrated to the desired concentration [protein concentration was determined using the Bio-Rad DC protein assay (Bio-Rad, Hercules, CA, USA)]. The purified proteins were then snap-frozen in small aliquots using liquid nitrogen and stored at −80°C.

For the phase separation assays, the purified proteins were transferred onto a slide, mixed with 0.2 M MES buffer (pH 6.5) or Hepes buffer (pH 7.5) to achieve an optimal salt concentration, and imaged on a Zeiss LSM880 confocal laser microscope at the following excitation wavelengths: GFP at 488 nm, mCherry at 564 nm, and bright field between 300 and 900 nm.

### Protoplast and plant fluorescence microscopy

Sterile NPB and 93-11 seedlings were grown on MS medium supplemented with 3% sucrose in a growth chamber with a 16-hour 30°C:8-hour 25°C, day:night, light:dark cycle for 10 days for subcellular localization assays.

Plasmids used for subcellular localization of rice mesophyll protoplasts were constructed using *163GFP* and *163mCherry* vectors. The coding sequences of *FLO6^jap^* and *FLO6^ind^* and *GBSS1^b^* and *GBSS1^a^* were amplified by PCR and recombined into *163GFP* and *163mCherry* respectively, resulting in *FLO6^jap^-GFP* and *FLO6^ind^-GFP*, and *GBSS1^b^-mCherry* and *GBSS1^a^-mCherry*. All the used primers are listed in table S5.

Mesophyll protoplasts were isolated from the leaf sheath of 10-day-old sterile NPB or 93-11 seedlings, and plasmids were transferred into protoplasts using the polyethylene glycol-calcium–mediated method and incubated for 16 hours in the dark at room temperature to allow for transient expression (*34*). For imaging of transformed protoplasts, samples were collected by centrifugation at room temperature and transferred on a slide. For imaging of homozygous transgenic plants expressing FLO6*^jap^*-GFP, FLO6*^ind^*-GFP, or FLO6*^jap^*_dIDR2_-GFP driven by the FLO6*^jap^* native promoter, and/or expressing GBSS1^b^-mCherry driven by the *GBSS1^b^* native promoter, hand-cut tissue slices of 15-DAP endosperm of transgenic plants were soaked in phosphate-buffered saline buffer on a slide. All the samples were then imaged on a Zeiss LSM880 confocal laser microscope or a high-sensitivity structured illumination microscope (HiS-SIM) at the following excitation wavelength: GFP at 488 nm, mCherry at 564 nm, and bright field between 300 and 900 nm.

### Protein fluorescence labeling assay

Purified FLO6*^jap^*_dIDR1_ and FLO6*^jap^*_dIDR2_ and FLO6-S-IDR2, and GBSS1^b^ and GBSS1^a^ were used to prepare the fluorescence-labeled proteins. Ultrafiltration was used for buffer exchange and to concentrate purified protein into a Hepes-based buffer [50 mM Hepes-NaOH (pH 7.5), 500 mM NaCl, 1 mM EDTA, and 1 mM dithiothreitol]. The protein conjugation assay was performed using the AbFluor 488 or AbFluor 594 Labeling Kit (Abbkine Scientific, KTL0520 and KTL0540) according to the manufacturer’s instructions.

### Quantification of condensate properties and FRAP assay

Condensate properties, including size and number, were evaluated in the 66.56 μm–by–66.56 μm field with ImageJ software. For the calculation of size and number, condensate areas that were smaller than 0.1 μm^2^ were excluded due to the resolution limit of images.

For the in vivo experiments, FRAP of FLO6 alleles/variant-GFP condensates in 15-DAP endosperm was performed on a Nikon AX confocal laser microscope with NSPARC mode. To ensure consistency in the FRAP analysis, target condensates were carefully selected based on the following criteria: location within the same focal plane, exhibiting clear morphology, having a similar cross-sectional area, and showing clear adherence to starch granules. A region of a FLO6-GFP condensate was bleached using a 488-nm laser pulse (three iterations, 100% intensity). Recovery was recorded every 7 s for a total of 120 s after bleaching, and at least 10 independent condensates (*n* = 10) were analyzed for each FLO6 allele/variant. In vitro FRAP experiments were performed with purified proteins using a Zeiss LSM880 confocal laser microscope with Airyscan (AxioObserver 7) mode using a ×63/1.40–numerical aperture oil objective. Recovery was recorded for a total of 90 s after bleaching. For all FRAP experiments, recoveries were measured as fluorescence intensity of photobleached area normalized to the intensity of the same area before bleaching. Analysis of the recovery curves was performed with Zeiss ZEN 2 (blue edition) software or ImageJ and Origin 2021 software.

### Chemical treatments

To evaluate the phase separation property of FLO6 in vivo, 1,6-hexanediol treatment on FLO6 condensate of 15-DAP endosperm of *FLO6^jap^p:FLO6^jap^-GFPcomp* grains was carried out. Briefly, the hand-cut tissue slices of 15-DAP endosperm were soaked in 5% 1,6-hexanediol solution on a slide, and transferred to a HiS-SIM and recorded every 30 s for a total of 10 min at the following excitation wavelength: GFP at 488 nm and bright field between 300 and 900 nm.

For 1,6-hexanediol or NaCl treatment, concentration of purified FLO6-GFP proteins was first determined using the Bio-Rad DC protein assay (Bio-Rad, Hercules, CA, USA). Then, similar amounts of purified proteins were mixed with 1,6-hexanediol or NaCl solution at different concentrations and pH, respectively. The mixed samples were observed with a Zeiss LSM880 with Airyscan (AxioObserver 7) mode confocal laser microscope at the following excitation wavelengths: GFP at 488 nm and bright field between 300 and 900 nm.

### Yeast two-hybrid assay

The *Saccharomyces cerevisiae* strain AH109 (*MAT*α, *trp1-901*, *leu2-3*, *112*, *ura3-52*, *his3-200*, *gal4*Δ, *gal80*Δ, *LYS2::GAL1UAS-GAL1TATA-HIS3*, *MEL1 GAL2UAS-GAL2TATA-ADE2*, and *URA3::MEL1UAS-MEL1TATA-lacZ*) yeast cell cultures were grown at 28°C in standard adenine-supplemented YPD and dropout mediums. Plasmids used for yeast two-hybrid assay were constructed using *pGBKT7* and *pGADT7* vectors. The coding sequences of *FLO6^jap^* and *FLO6^ind^*, *GBSS1^b^* and *GBSS1^a^*, and *OsGLK1^jap^* (LOC_Os06g24070) were amplified by PCR and recombined into *pGADT7* and *pGBKT7*, respectively, resulting in specific plasmids for yeast two-hybrid assay. All the used primers are listed in table S5. Plasmids were transformed into the yeast strain AH109 in combination. Yeast transformants were grown on synthetic dropout medium containing 10 μg/liter X-α-Gal (5-bromo-4-chloro-3-indolyl-a-d-galactopyranoside) without tryptophan, leucine, and histidine at 28°C for 4 days.

### MST assay

MST assays were performed to determine the binding affinities between FLO6 and GBSS1. Purified GBSS1^b^-GFP-SUMO (600 nM) or purified GBSS1^a^-GFP-SUMO (100 nM) was mixed with purified FLO6*^jap^*-MBP or FLO6*^ind^*-MBP at a concentration ranging from 0.02 to about 700 μM in Hepes buffer in combination. Samples were first centrifuged at 13,000 rpm at 4°C for 10 min and then loaded into capillaries (Monolith NT.115 Standard Treated Capillaries, MO-K022). All measurements were performed using a Monolish NT.115 device (NanoTemper Technologies) at medium MST power and 80% light-emitting diode power. The *K*_d_ Fit function of the NanoTemper Analysis Software (Version 1.5.41) was used to fit the curve and calculate the value of the dissociation constant (*K*_d_).

### Total protein preparation and immunoblot analysis

Total protein was extracted from seedling or developing endosperm of rice. Briefly, samples were homogenized in an ice-cold extraction buffer [50 mM tris-HCl (pH 7.5), 100 mM NaCl, 10% (v/v) glycerol, 1 mM phenylmethylsulfonyl fluoride, 1% (v/v) Triton X-100, and protease inhibitors (1 μg/ml of aprotinin, leupeptin, and pepstain, freshly added)] on ice. After incubation for 1 hour at 4°C with shaking, the supernatant was collected by centrifugation at 12,000*g* at 4°C for further analyses.

Protein concentrations in the supernatant were first determined using the Bio-Rad DC protein assay (Bio-Rad, Hercules, CA, USA). For immunoblot analysis, immunoblot assays were carried out. Briefly, the supernatant was mixed with SDS buffer and boiled for 5 min, and separated by SDS-PAGE and transferred to nitrocellulose membranes. The membranes were incubated with specific primary antibodies, and signals from secondary conjugated antibodies were detected by enhanced chemiluminescence. Anti-GBSS1^b^ rabbit polyclonal antibody was purchased from PhytoAB company (San Francisco, USA); anti-FLO6*^jap^* rabbit polyclonal antibody was prepared by Immunogen Corporation (Wuhan, China) using purified FLO6*^jap^* (purity of >95%) as antigen.

### Measurement of GBSS1 activity

In vivo and in vitro GBSS1 activity was measured with a kit (GBSS Activity Assay Kit, Solarbio, BC3290) according to the operation manual. In this assay, GBSS catalyzes the transfer of glucose from adenosine 5′-diphosphate (ADP)–glucose to a starch primer (glucan), releasing ADP. The generated ADP is subsequently used in a coupled enzymatic system involving pyruvate kinase, hexokinase, and 6-phosphogluconate dehydrogenase, which ultimately reduces nicotinamide adenine dinucleotide phosphate (NADP^+^) to NADPH. Consequently, the increase in NADPH absorbance at 340 nm is directly proportional to GBSS1 activity.

For the in vivo assay, 0.1 to 0.2 g of endosperm was isolated from 15-DAP developing grains and homogenized in 1.5 ml of cold extraction buffer [50 mM Hepes-NaOH (pH 7.4), 4 mM MgCl_2_, 2 mM EDTA, 50 mM 2-mercaptoethanol, and 12.5% (v/v) glycerol] on ice. The homogenate was centrifuged at 10,000*g* for 10 min at 4°C. To eliminate interference from soluble starch synthases, the supernatant was discarded, and the starch pellet was continuously washed with the extraction buffer before final resuspension. Furthermore, potential interference from GBSS2 was excluded because GBSS1 is predominantly expressed in the endosperm, whereas GBSS2 is expressed in leaf tissues. A *gbss1* KO mutant was also included as a control to validate the specificity of the assay. To verify that any observed differences in activity were not merely due to altered protein expression, GBSS1 protein abundance in the corresponding samples was examined in parallel. The activity of GBSS1^b^ was measured with an ultraviolet spectrophotometer (U-T5C, MAPADA, Shanghai) by quantifying the amount of NADPH at 340 nm, which was proportional to ADP produced by GBSS1^b^. The activity of GBSS1^b^ was calculated according to the formula mentioned in the operation manual. For the in vitro assay, the purified FLO6 and GBSS1 proteins (*m*_FLO6_:*m*_GBSS1_ = 2.5:1) were first mixed in 10% dextran solution in combination for 5 min to generate protein phase separation and recruitment. Then, the protein mixture was used for measuring GBSS1 activity according to the procedure mentioned above.

### Analyses of the starch parameters and physicochemical properties of endosperm

The starch content of the rice grains was measured with an amylum content assay kit (D799325-0050, Sangon Biotech), and amylose content was assessed with an amylose content assay kit (D799147-0100, Sangon Biotech), according to the manufacturer’s protocol. Briefly, the amylose quantification uses an iodine-potassium iodide colorimetric method coupled with a dual-wavelength detection system. By measuring the absorbance at both 550 and 485 nm, this method mathematically minimizes the background interference from the amylopectin-iodine complex, allowing for the specific quantification of amylose. To validate the specificity of this assay, grains from a *waxy* mutant (*GBSS1^b^-KO12*) were included as a control. For the gelatinization temperature analysis, 5 mg of rice powder was placed in an aluminum sample cup, mixed with 10 μl of distilled water and sealed. The samples were analyzed by a DSC (DSC 1; Mettler Toledo, Zurich, Switzerland) following the operation manual.

### Observation of granules by scanning electron microscopy

To observe the starch granules on a cross-section of endosperm of rice grains, brown rice was frozen in liquid nitrogen for 2 min and broken in half using tweezers. The prepared samples were mounted on specimen stubs, sputter-coated with gold, and observed under a Nova NanoSEM 450 (FEI, USA).

### Analyses of geographic and subpopulation distribution of SNP^3194^

*FLO6* SNP^3194^ dataset (containing 339 tropical *japonica*, 108 subtropical *japonica*, 262 temperate *japonica*, 71 *japx*, 189 *ind1A*, 177 *ind1B*, 210 *ind2*, 431 *ind3*, and 496 *indx*) was downloaded from the Rice SNP-Seek Database (snp-seek.irri.org/) (table S2). *FLO6* SNP^3194^ of 366 wild rice individuals including two *O. rufipogon* (Ruf1 and Ruf2) and two *O. nivara* (Niv1 and Niv2) lineages were searched with BLAST (table S2). Data were used for examining geographic and subpopulation distribution of SNP^3194^.

### Analyses of agricultural traits

Field experiments were conducted in paddy fields from May to September in Wuhan (118°E, 32°N) from 2022 to 2024 and from December to the next April in Lingshui (110°E, 18°N) from 2024 to 2025. During the field experiments, the plant height was determined as the height of the main tiller at the filling stage.

### Analyses of the relation between *Wx* alleles and grain amylose content in *japonica* and *indica* rice

Forty-six *japonica* accessions with *Wx^b^*, 14 *indica* accessions with *Wx^b^*, and 26 *indica* accessions with *Wx^a^*, which were reported previously (*19*, *21*, *22*), were selected to analyze the relation between *Wx* alleles and grain amylose content in different rice subspecies (table S4).

### Statistical analyses

Statistical analyses to determine significant differences among the wild-type NPB and mutant and transgenic lines and base-edited lines were performed by two-tailed Student’s *t* test using Microsoft Excel or one-way analysis of variance (ANOVA) analysis followed by the pairwise post hoc Tukey test using Origin 2021 software. *P* values smaller than 0.05 or 0.01 were considered statistically significant or extremely significant.
