# Supplementary material for: Differential condensation of FLO6 influences grain amylose synthesis in rice
Source: Sci Adv. 2026 Aug 14;12(33):eaec8768. doi: 10.1126/sciadv.aec8768 (PMC13475482; doi:10.1126/sciadv.aec8768)
Supplement: Supplementary file 1 [file sciadv.aec8768_sm.pdf]

Science Advances
AAAS

Supplementary Materials for

Differential condensation of FLO6 influences grain amylose synthesis in rice

Fei Liu *et al.*

Corresponding author: Min Ouyang, ouyangmin@mail.hzau.edu.cn

Sci. Adv. **12**, eaec8768 (2026)
DOI: 10.1126/sciadv.aec8768

The PDF file includes:

Figs. S1 to S8
Legends for tables S1 to S5

Other Supplementary Material for this manuscript includes the following:

Tables S1 to S5

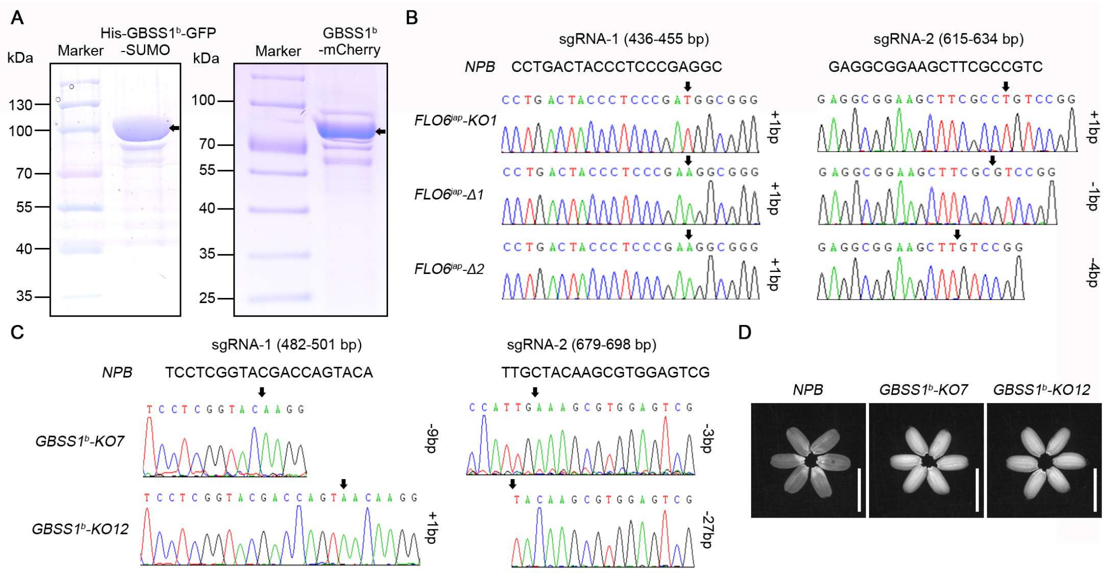

Fig. S1. Purification of His-GBSS1^{b}-GFP-SUMO and GBSS1^{a}-mCherry and creation of the *FLO6^{jap}-KO* and *GBSS1^{b}-KO* mutants. (A) Coomassie stained SDS-PAGE gel of the purified His-GBSS1^{b}-GFP-SUMO and GBSS1^{b}-mCherry. Black arrows indicate the target proteins. **(B and C)** DNA sequencing chromatograms of *FLO6^{jap}* in homozygous *FLO6^{jap}* mutants, and *GBSS1^{b}* in homozygous *GBSS1^{b}* mutants, generated in the *NPB* background. Black arrows indicate the regions with nucleotide insertions or deletions. All indicated positions correspond to the respective genomic DNA (gDNA) sequences, and all these mutations are located within the exonic regions. Specifically, *FLO6^{jap}-KO1* contains 1-bp insertions (+T) between positions 452/453 and 631/632. *FLO6^{jap}-Δ1* carries a 1-bp insertion (+A) between positions 452/453 and a 1-bp deletion (-C) at position 631. *FLO6^{jap}-Δ2* features a 1-bp insertion (+A) between positions 452/453 and a 4-bp deletion at positions 628–631. For the *GBSS1^{b}* mutants, *GBSS1^{b}-KO7* displays a 9-bp deletion (positions 492–500) and three single-base deletions at positions 682, 683, and 685. *GBSS1^{b}-KO12* contains a 1-bp insertion (+A) between positions 498/499 and a 27-bp deletion at positions 656–682. **(D)** Representative grains from *NPB* and *GBSS1^{b}-KO* mutants. Scale bars, 1 cm.

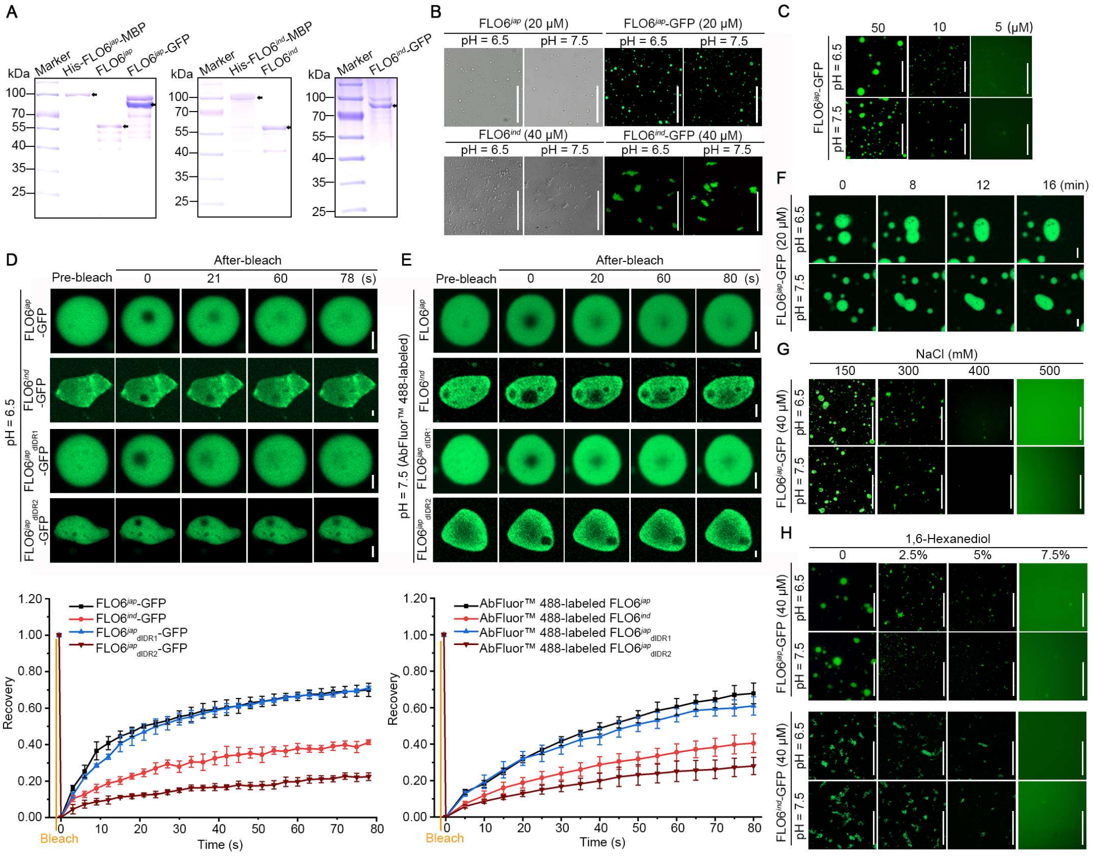

Fig. S2. FLO6^{jap} and FLO6^{ind} phase separate into different condensates *in vitro*. (**A**) Coomassie stained SDS-PAGE gel of purified His-tag-cleaved FLO6^{jap} and FLO6^{ind}, and 6×His-tagged FLO6^{jap}-GFP and FLO6^{ind}-GFP. Black arrows indicate the target proteins. **(B)** *In vitro* phase separation assays of purified His-tag-cleaved FLO6^{jap} and FLO6^{ind}, and 6×His-tagged FLO6^{jap}-GFP and FLO6^{ind}-GFP at different pH values and indicated protein concentrations. Scale bars, 50 μm. **(C)** Droplet formation of 6×His-tagged FLO6^{jap}-GFP at varying protein concentrations. Scale bars, 50 μm. **(D** and **E)** Fluorescence recovery after photobleaching (FRAP) of purified 6×His-GFP-tagged FLO6 variants at pH 6.5 **(D)**, and AbFluor™ 488 labeled His-tag-cleaved FLO6 variants at pH 7.5 **(E)**. Corresponding fluorescence recovery curves are shown below. Data are presented as means ± SD (n = 5). Scale bar, 2 μm. **(F)** Fusion events of purified 6×His-tagged FLO6^{jap}-GFP condensates at different pH values and indicated protein concentrations. Scale bar, 2 μm. **(G)** Phase separation of purified 6×His-tagged FLO6^{jap}-GFP in the presence of varying concentrations of NaCl at different pH values. Protein concentrations are as indicated. Scale bars, 50 μm. **(H)** Phase separation of purified 6×His-tagged FLO6^{jap}-GFP and FLO6^{ind}-GFP in the presence of varying concentrations of 1,6-hexanediol at different pH values. Protein concentrations are as indicated. Scale bars, 50 μm.

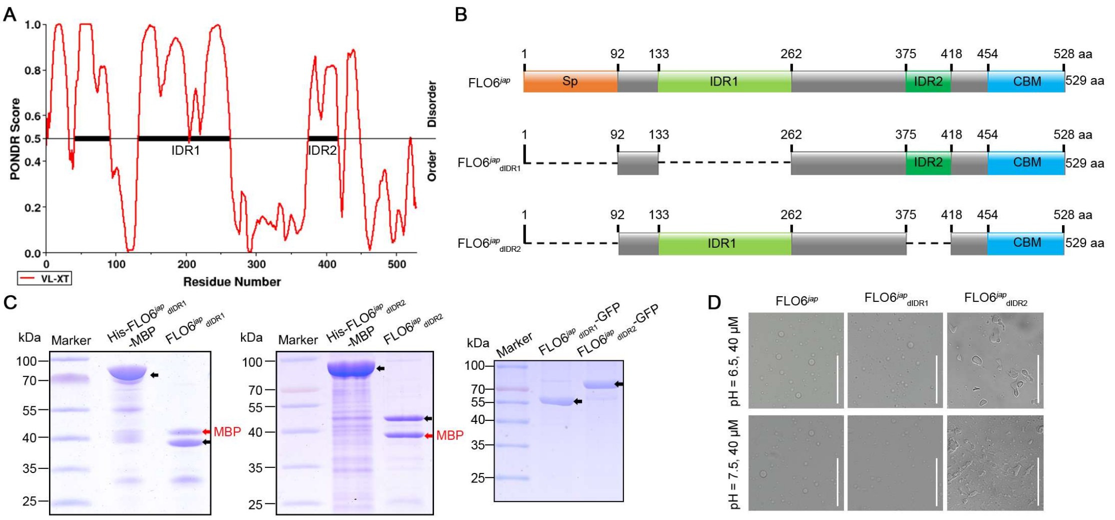

Fig. S3. Expression and purification of the IDR-deleted FLO6^{jap}. (A) Prediction of intrinsically disordered region (IDR) of FLO6^{jap} by PONDR (http://www.pondr.com). The most disordered regions of the FLO6^{jap} (residues 42-92 (located in the signal peptide), 133-262 (named IDR1), 375-418 (named IDR2)) are marked with bold black lines. **(B)** Protein structure of FLO6^{jap}, FLO6$^{jap}_{dIDR1}$ and FLO6$^{jap}_{dIDR2}$. Sp represents signal peptide, CBM represents carbohydrate binding module. **(C)** Coomassie stained SDS-PAGE gel of the purified His-tag-cleaved FLO6$^{jap}_{dIDR1}$ and FLO6$^{jap}_{dIDR2}$, and 6×His-tagged FLO6$^{jap}_{dIDR1}$-GFP and FLO6$^{jap}_{dIDR2}$-GFP. Black arrows indicate the target proteins; red arrows indicate the MBP tag. **(D)** *In vitro* phase separation assays of purified FLO6^{jap}, FLO6$^{jap}_{dIDR1}$ and FLO6$^{jap}_{dIDR2}$ at different pH values and the indicated protein concentrations. Scale bars, 50 μm.

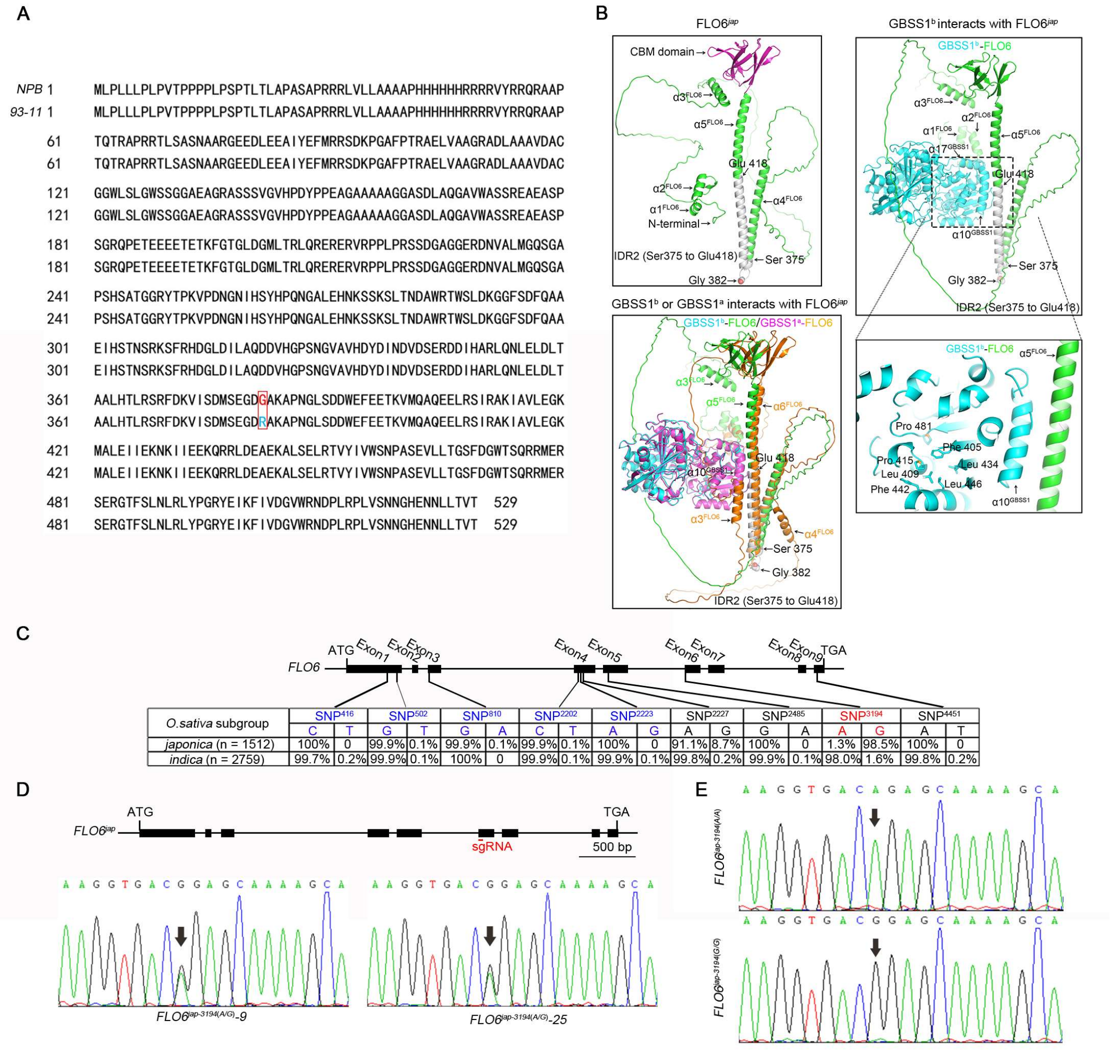

Fig. S4. Creation and identification of the SNP^{3194}-edited *NPB* plants in the first (T0) and second (T1) generation. (A) Amino acid sequence comparison between FLO6^{jap} (from *NPB*) and FLO6^{ind} (from *93-11*). Red box indicates the different amino acid. **(B)** Structural modeling of the FLO6-GBSS1 interaction predicted by AlphaFold3. Top left: Predicted individual structure of FLO6. The CBM domain is shown in magenta, the IDR2 motif (residues 375–418) in gray, and the remainder of the protein in green. Arrows indicate the positions of all α-helices, the start of IDR2, and the Gly382 residue. Top right: Close-up of the interaction interface between FLO6 and GBSS1^{b}. GBSS1^{b} is colored cyan, while the IDR2 of FLO6 motif and the rest of the protein are shown in gray and green, respectively. Arrows highlight the α-helices contributing to the interface. The hydrophobic core stabilized by P415 of GBSS1^{b} and the primary FLO6-GBSS1 interaction helix are enclosed in dashed boxes (detailed in the bottom right panel). Bottom right: Detailed view of the hydrophobic core centered around P415. Interacting residues forming the core are represented as sticks. Bottom left: Structural superposition of the FLO6-GBSS1^{b} and FLO6-GBSS1^{a} complexes. In the FLO6-GBSS1^{b} module, FLO6 is green (with IDR2 in gray) and GBSS1^{b} is cyan. In the FLO6-GBSS1^{a} module, FLO6 is shown in orange and GBSS1^{a} in magenta. Arrows indicate key interface helices and regions exhibiting structural divergence between the two complexes. **(C)** Allele frequency of nine SNPs in *FLO6* exons in analyzed *japonica* and *indica* populations. Data are from

RiceVarMap2 database. SNP in blue or red indicates SNP in the IDR1 or IDR2 of *FLO6.* **(D)** Schematic view indicating a sgRNA (red line) for $FLO6^{jap}$ and $FLO6^{jap\text{-}3194(G/A)}$ in *NPB*. DNA sequencing chromatograms of the base-edited region in $FLO6^{jap}$ from two edited plants are shown below, and black arrows indicate the SNP^{3194}. **(E)** DNA sequencing chromatograms of base-edited region in $FLO6^{jap}$ of $FLO6^{jap\text{-}3194(A/A)}$ and $FLO6^{jap\text{-}3194(G/G)}$ plants in T1 generation. Black arrows indicate the SNP^{3194}.

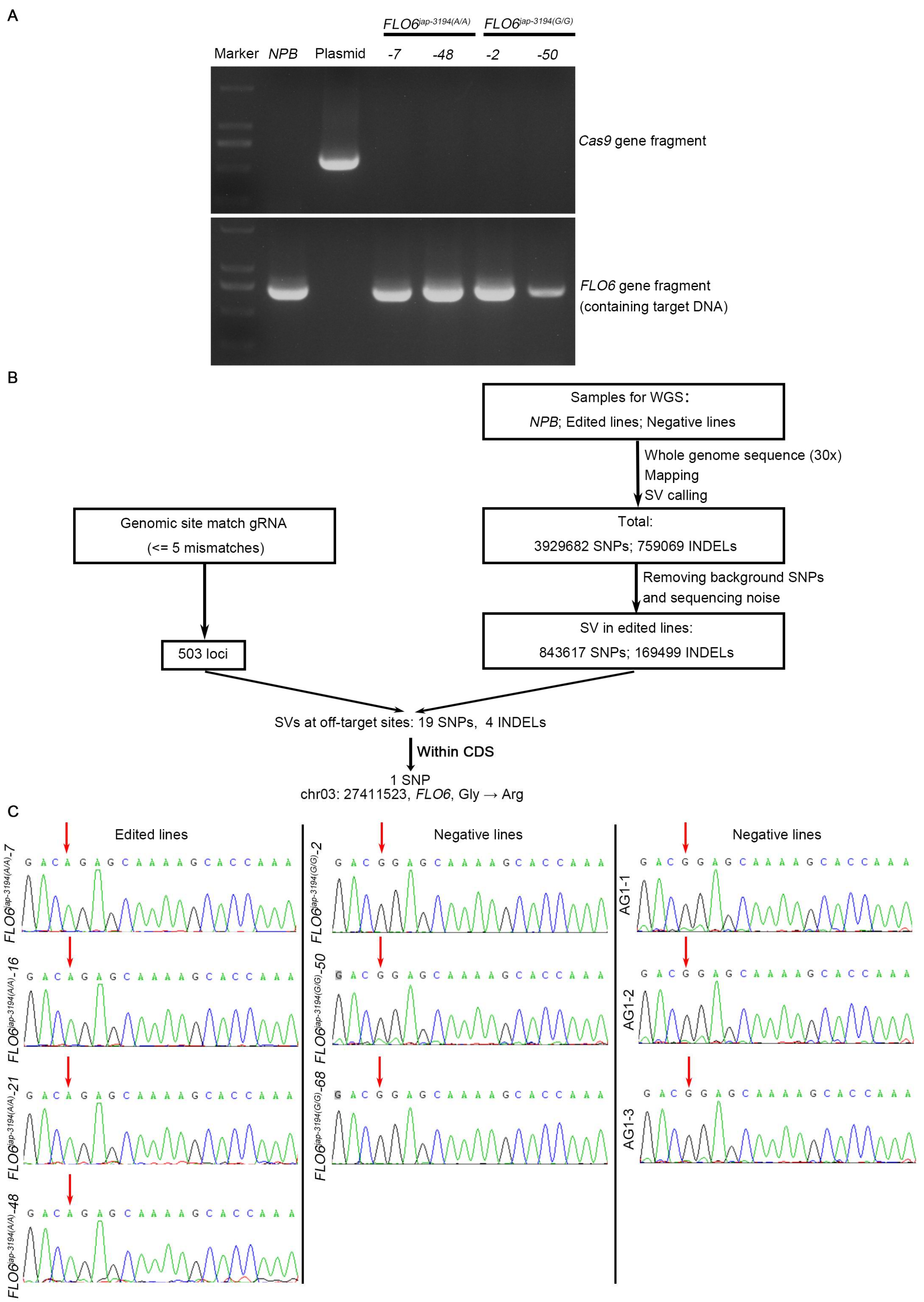

Fig. S5. Identification of Cas9-free lines and whole-genome off-target analysis of *FLO6* SNP^{3194}-edited plants in the T2 generation.

(A) PCR detection of the Cas9 transgene in edited *NPB* plants. The absence of bands indicates *Cas9*-free lines. Amplification of a *FLO6* fragment served as an internal DNA quality control. **(B)** Flowchart of the whole-genome sequencing (WGS) strategy for off-target analysis. WGS

was performed on 14 T2-generation lines: four wild-type controls (*NPB1*, *NPB2*, *NPB3*, and *NPB4*); three unedited transgenic lines (*AG1-1*, *AG1-2*, and *AG1-3*); four homozygous edited lines ($FLO6^{jap\text{-}3194(A/A)}$*-7*, $FLO6^{jap\text{-}3194(A/A)}$*-16*, $FLO6^{jap\text{-}3194(A/A)}$*-21*, and $FLO6^{jap\text{-}3194(A/A)}$*-48*), and three null segregant lines ($FLO6^{jap\text{-}3194(G/G)}$*-2*, $FLO6^{jap\text{-}3194(G/G)}$*-50*, and $FLO6^{jap\text{-}3194(G/G)}$*-68*). Notably, both the edited and null segregant lines were derived from the segregation of a single T0 heterozygote, with the latter serving as isogenic controls. The detailed results of the off-target analysis are presented in Table S3. **(C)** DNA sequencing chromatograms of the $FLO6^{jap}$ target region in T2 plants. The chromatograms confirm the genotypes of the WGS samples, including the four homozygous edited lines ($FLO6^{jap\text{-}3194(A/A)}$) and the negative controls, which comprise three null segregant lines ($FLO6^{jap\text{-}3194(G/G)}$) and three unedited transgenic lines (*AG1*). Red arrows indicate the SNP^{3194} locus.

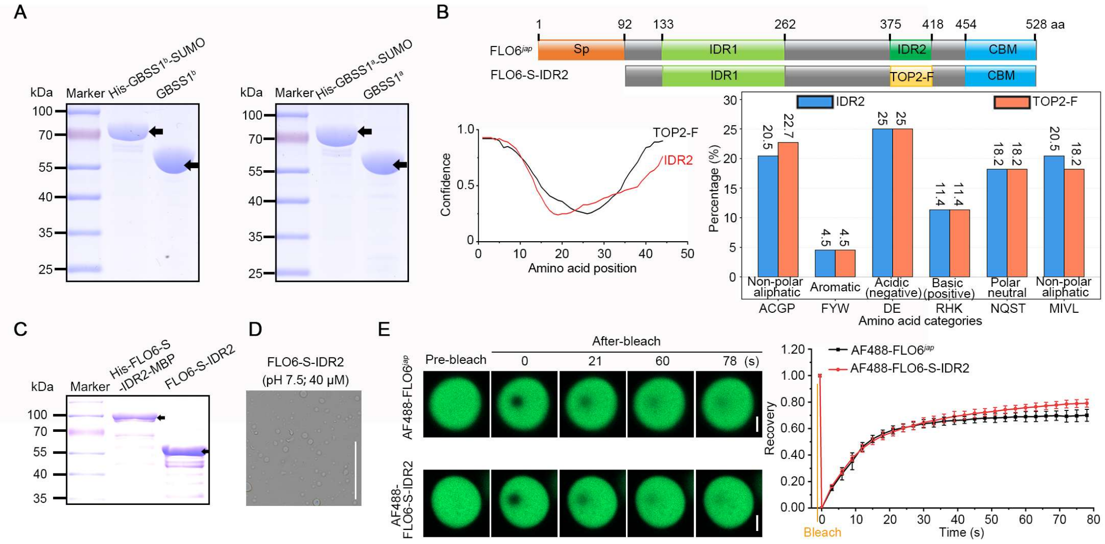

Fig. S6. Purification of GBSS1^b and GBSS1^a and evaluation of the phase separation of recombinant FLO6-S-IDR2. (A) Coomassie stained SDS-PAGE gel of the purified His-tag-cleaved GBSS1^b and GBSS1^a. Black arrows indicate the target proteins. **(B)** Protein structure of FLO6^{jap} and FLO6-S-IDR2. The disorder confidence scores of the amino acids in IDR2 and TOP2-F are shown in the bottom left corner. The disorder probability of each residue was predicted using PrDOS (https://prdos.hgc.jp/cgi-bin/top.cgi), and the comparison of amino acid composition between IDR2 and TOP2-F is shown in the bottom right corner. **(C)** Coomassie stained SDS-PAGE gel of the purified His-tag-cleaved FLO6-S-IDR2. Black arrows indicate the target proteins. **(D)** *In vitro* phase separation assay of purified FLO6-S-IDR2 at pH 7.5 and the indicated protein concentrations. Scale bars, 50 μm. **(E)** FRAP analysis of AbFluor™ 488-labeled FLO6-S-IDR2 at pH 7.5 and the indicated protein concentrations. Scale bar, 2 μm. Plot of fluorescence recovery is shown on the right. Values are means ± SDs (n = 5).

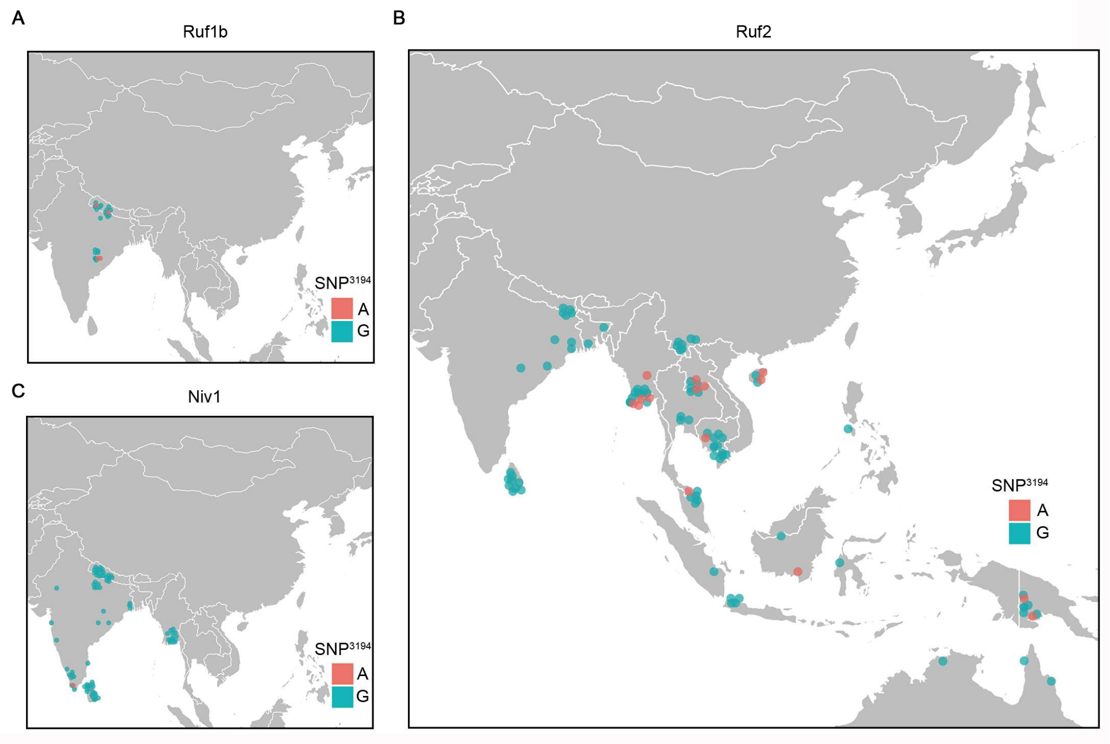

Fig. S7. Geographic distribution of SNP^{3194} in *FLO6* in Ruf1b, Ruf2 and Niv1. (A–C) Geographic distribution of SNP^{3194} in *FLO6* in Ruf1b **(A)**, Ruf2 **(B)** and Niv1 **(C)**.

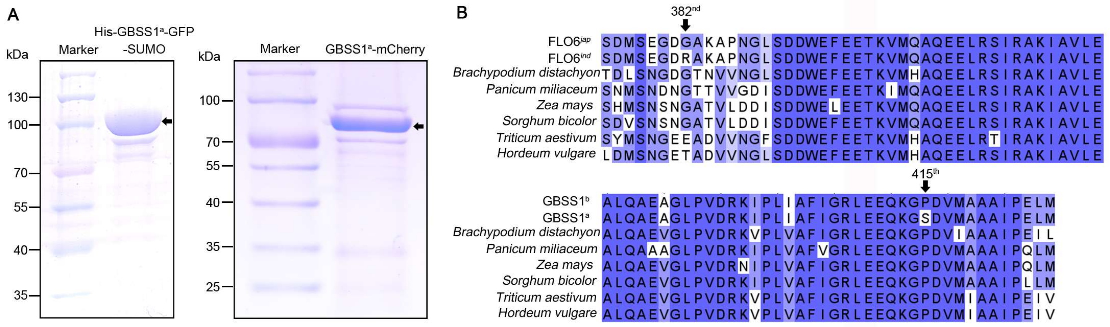

Fig. S8. Purification of His-GBSS1ᵃ-GFP-SUMO and GBSS1ᵃ-mCherry and alignments of the 382nd of FLO6 and the 415th of GBSS1 in *Gramineous* plants. (A) Coomassie stained SDS-PAGE gel of the purified His-GBSS1ᵃ-GFP-SUMO and GBSS1ᵃ-mCherry. Black arrows indicate the target proteins. **(B)** Alignments of the 382nd amino acid of FLO6, and the 415th of GBSS1 in *Gramineous* plants with Jalview (Version 2.11.5.1). Black arrows indicate the target amino acids above the sequences.

Supplementary Tables

Table S1. Candidate interactors of GBSS1 in Y2H

Table S2. Rice accessions used for analysis of the type of the 3194th SNP of FLO6

Table S3 Off-target analysis by WGS of base-edited plants in T2

Table S4. Rice accessions used for analysis of the relation between Wx and grain amylose content

Table S5. Primers used in this study
